# NUDT7 Loss Promotes *Kras^G12D^* CRC Development

**DOI:** 10.3390/cancers12030576

**Published:** 2020-03-02

**Authors:** Jinsoo Song, Sujeong Park, Jinjoo Oh, Deokha Kim, Ji Hyun Ryu, Won Cheol Park, In-Jeoung Baek, Xi Cheng, Xin Lu, Eun-Jung Jin

**Affiliations:** 1 Department of Biological Sciences, College of Natural Sciences, Wonkwang University, Iksan, Chunbuk 54538, Korea; songjinsoo85@wku.ac.kr (J.S.); kasgami@wku.ac.kr (S.P.); wlswn9408@gmail.com (J.O.); ejrgk29@wku.ac.kr (D.K.); 2 Integrated Omics Institute, Wonkwang University, Iksan, Chunbuk 54538, Korea; 3 Department of Carbon Convergence Engineering, College of Engineering, Wonkwang University, Iksan, Chunbuk 54538, Korea; jhryu4816@wku.ac.kr; 4 Department of Surgery, Wonkwang University School of Medicine, Iksan, Chunbuk 54538, Korea; parkwc@wku.ac.kr; 5 Asan Institute for Life Sciences, University of Ulsan College of Medicine, Seoul, 05505, Korea; ijbaek@amc.seoul.kr; 6 Department of Biological Sciences, Boler-Parseghian Center for Rare and Neglected Diseases, Harper Cancer Research Institute, University of Notre Dame, Notre Dame, IN 46556, USA; drchengxi@126.com; 7 Department of General Surgery, Ruijin Hospital, Shanghai Jiao Tong University School of Medicine, Shanghai, 200025, China

**Keywords:** peroxisome, colorectal cancer, peroxisomal coenzyme A diphosphatase NUDT7 (NUDT7), palmitic acid, β-catenin

## Abstract

Studies have suggested that dysregulation of peroxisomal lipid metabolism might play an important role in colorectal cancer (CRC) development. Here, we found that *Kras^G12D^*-driven CRC tumors demonstrate dysfunctional peroxisomal β-oxidation and identified *Nudt7* (peroxisomal coenzyme A diphosphatase NUDT7) as one of responsible peroxisomal genes. In *Kras^G12D^*-driven CRC tumors, the expression level of *Nudt7* was significantly decreased. Treatment of azoxymethane/dextran sulfate sodium (AOM/DSS) into *Nudt7* knockout (*Nudt7^−/−^*) mice significantly induced lipid accumulation and the expression levels of CRC-related genes whereas xenografting of *Nudt7*-overexpressed LS-174T cells into mice significantly reduced lipid accumulation and the expression levels of CRC-related genes. Ingenuity pathway analysis of microarray using the colon of *Nudt7^−/−^* and *Nudt7^+/+^* mice treated with AOM/DSS suggested Wnt signaling as one of activated signaling pathways in *Nudt7^−/−^* colons. Upregulated levels of β-catenin were observed in the colons of *Kras^G12D^* and AOM/DSS-treated *Nudt7^−/−^* mice and downstream targets of β-catenin such as *Myc*, *Ccdn1,* and *Nos2*, were also significantly increased in the colon of *Nudt7^−/−^* mice. We observed an increased level of palmitic acid in the colon of *Nudt7^−/−^* mice and attachment of palmitic acid-conjugated chitosan patch into the colon of mice induced the expression levels of β-catenin and CRC-related genes. Overall, our data reveal a novel role for peroxisomal *NUDT7* in *Kras^G12D^*-driven CRC development.

## 1. Introduction

Colorectal cancer (CRC) is one of the most common cancers, with over one million new cases diagnosed worldwide each year. It is the third most common malignancy and the second most common cause of cancer mortality [1]. Tumorigenesis of CRC involves a multi-step process through the accumulations of genetic and epigenetic alterations. High-penetrance mutations of the mismatch repair genes (*Mlh1,* DNA mismatch repair protein Mlh1; *Msh2*, DNA mismatch repair protein Msh2; *Msh6*, DNA mismatch repair protein Msh6; *Pms2,* mismatch repair endonuclease Pms2; *Apc*, adenomatous polyposis coli protein; *Mutyh,* adenine DNA glycosylase; *Smad4,* SMAD family member 4; *Bmpr1A*, bone morphogenetic protein receptor type 1A; *Stk11/Lkb1,* serine-threonine kinase 11; *Pten,* phosphatase and tensin homolog; *Axin2,* axis inhibition protein 2; *Pole*, DNA polymerase epsilon, catalytic subunit; *Pold1,* DNA polymerase delta 1, catalytic subunit) have been reported as CRC predisposing factors [2]. Exome sequencing of common familial CRC suggested 11 novel candidate CRC susceptibility genes, including *Uaca*, *Sfxn4*, *Twsg1*, *Psph*, *Nudt7*, *Znf490*, *Prss37*, *Ccdc18*, *Pradc1*, *Mrpl3*, and *Akr1c4*, with rare truncating variants [3]. Although many molecular events have been identified, new molecules that play a role in this process remain to be discovered, and are crucial for the development of improved therapeutic approaches.

Currently, in CRC, drugs either targeting angiogenesis (bevacizumab, aflibercept, and ramucirumab) or targeting the epidermal growth factor receptor (EGFR; cetuximab and panitumumab) have been developed [4]. Cetuximab and panitumumab are both monoclonal antibodies directed against the extracellular domain of the EGFR and block the downstream RAS (Ras proto-oncogene, GTPase)-RAF (RF proto-oncogene serin/threonine-protein kinase)-MEK (mitogen-activated protein kinase kinase 1)-ERK signaling pathway [5,6]. However, the clinical response of CRC to anti-EGFR Abs is poor for tumors with the Kirsten rat sarcoma viral oncogene homolog (*Kras*) mutation mostly at codons 12, 13, or 61 [7,8], which are found in 40% of metastatic CRCs. Identifying the molecular pathogenic and regulatory processes of CRC with *Kras* mutation (*Kras^mut^*) will likely provide promising strategies for controlling *Kras^mut^* CRC. Thus, a deeper understanding of the molecular and genetic networks that control the initiation and progression of *Kras^mut^* CRC is essential.

Lipid metabolism, particularly fatty acid β-oxidation, is an essential process for cancer cell proliferation, differentiation, motility, and growth [9,10]. An appropriate ratio between saturated fatty acids (SFAs) and monounsaturated fatty acids (MUFAs) is required for proper membrane fluidity and cell function, and an increased amount of MUFAs has been observed in several cancers [11,12]. Stearoyl-CoA (Coenzyme A) desaturase-1 (SCD1), the rate-limiting enzyme converting SFAs into MUFAs, has been described to be upregulated in several types of human tumors and is known to be correlated with malignant transformation, proliferation, and survival of cancer cells [13]. Acyl-CoA synthetases (ACSs), the rate-limiting enzyme converting long chain fatty acids (LCFAs) to acyl-CoA, is also related to carcinogenesis [14]. A study demonstrated that the *Kras*-dependent regulation of lipid metabolism is a critical factor for lung tumorigenesis [15] and fatty acid-induced inflammatory mediators such as prostaglandin E2, leukotriene B4, interleukin 1β, and tumor necrosis factor α, are known to act as stimulatory factors for cancer cell growth and proliferation [16]. 

In mammalian cells, peroxisome is the main subcellular organelle involved in the β-oxidation pathway, a key pathway for the breakdown of fatty acids [17,18]. In peroxisomes, the first step of fatty acid oxidation is the conversion reaction of fatty acids into acyl-CoA. Peroxisomal β-oxidation mainly concerns very long chain fatty acids (VLCFAs, >C22) and branched fatty acids, as well as some prostaglandins and leukotrienes [19,20,21]. Even though the importance of peroxisome in the regulation of various cellular functions including lipid metabolism has emerged, our knowledge of the function and role of peroxisomes is limited.

In this study, we evaluate the importance of peroxisomal function in *Kras^G12D^* CRC and identify peroxisomal coenzyme A diphosphatase NUDT7 (NUDT7) as a potent tumor suppressor to restrict *Kras^G12D^* CRC progression. 

## 2. Results

### 2.1. Peroxisomal Dysfunction Is Responsible for Dysregulation of Lipid Metabolism in Kras^G12D^ CRC

**Figure 1:**
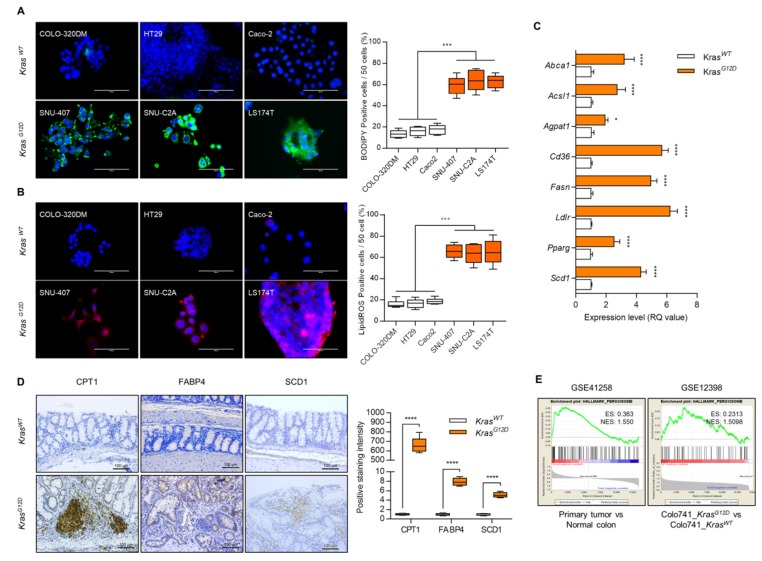
Dysfunction of lipid metabolism in *Kras^G12D^* colorectal cancer (CRC) cells. (**A**) BODIPY staining and (**B**) lipid reactive oxygen species (ROS) staining using *Kras^G12D^* and *Kras^WT^* cell lines. Positive cells were counted for every 50 cells in 3 different fields at 400× magnification. Results shown are representative of at least 3 independent experiments. Scale bars: 100 μm. (**C**) Expression level of genes involved in lipid metabolism in *Kras^G12D^* CRC cells and presented as the fold change of *Kras^WT^* CRC cells. *Rn18s* was used as an endogenous control. Results are representative of at least 3 independent experiments. (**D**) Immunohistochemical staining with CPT1, FABP4, and SCD1, and positive cells were counted (*n* = 4). Scale bars: 100 μm. (**E**) GSEA analysis using GEO datasets (CRC patient biopsy dataset, GSE41258 and *Kras^G12D^* transfected CRC cell line dataset, GSE12398). Values are presented as means + SD. A two-tailed Student’s *t*-test was used for statistical analysis. * *p* ≤ 0.05, *** *p* < 0.001, **** *p* < 0.0001.

To assess the alteration of lipid metabolism in *Kras^G12D^* CRC compared with *Kras^WT^* CRC, we analyzed lipid reactive oxygen species (ROS) and lipid accumulation in *Kras^G12D^* CRC cells (SNU-407, SNU-C2A, and LS-174T) and *Kras^WT^* cells (COLO-320DM, HT29, and Caco2). The number of BODIPY and lipid ROS-positive cells was significantly increased (average of 3.95-fold of *Kras^WT^* CRC cells and 3.77-fold of *Kras^WT^* CRC cells in BODIPY and lipid ROS-positive cells, respectively) in *Kras^G12D^* CRC cells compared with those in *Kras^WT^* CRC cells (Figure 1A,B). The expression levels of genes in lipid metabolism such as *Abca1*, *Acsl1*, *Agpat1*, *Cd36*, *Fasn*, *Ldlr*, *Pparg*, and *Scd1*, were dramatically increased in *Kras^G12D^* CRC cells (Figure 1C). We also observed the upregulation of carnitine palmitoyltransferase-1 (CPT1) (662.85-fold of *Kras^WT^* CRC tumor), fatty acid binding protein 4 (FABP4) (7.87-fold of *Kras^WT^* CRC tumor), and SCD1 (5.1-fold of *Kras^WT^* CRC tumor) in *Kras^G12D^* CRC tumors (*n* = 4) compared with *Kras^WT^* CRC tumors (Figure 1D). Gene set enrichment analysis (GSEA) using GSE41258 (186 primary tumors compared with 54 normal colons) and GSE12398 (*Kras^G12D^* transfected Colo741 cells compared with *Kras^WT^* transfected Colo741 cells), showed decreased expression of peroxisome-related genes in *Kras^G12D^* CRC tumors (Figure 1E).

**Figure 2:**
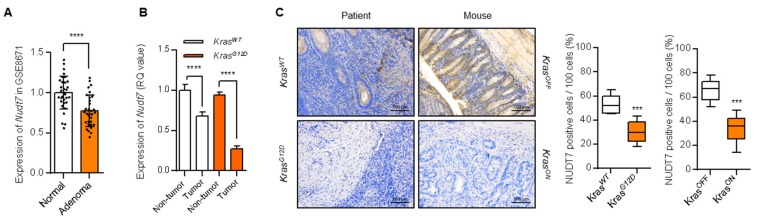
Decreased level of peroxisomal *Nudt7* is observed in *Kras^G12D^* CRC dysregulation. (**A**) Expression level of *Nudt7* in CRC tumors using GSE8671 dataset and presented as the fold change compared with normal. (**B**) Expression level of *Nudt7* in tumors or adjacent non-tumor areas of *Kras^G12D^* and *Kras^WT^* CRC. *Rn18s* was used as an endogenous control (*n* = 3). (**C**) NUDT7 expression was analyzed via immunohistochemistry using tamoxifen-inducible *Villin-CreER^T2^; Apc^f/f^; Trp53^f/f^; tetO-LSL-Kras^G12D^* mice (*Kras^OFF^* and *Kras^ON^*) and human patients (*Kras^WT^* and *Kras^G12D^*). Positive cells were counted for every 100 cells in 3 different fields at 200× magnification. Results are representative of at least 3 independent experiments. Scale bars: 100 μm. Values are means + SD. An unpaired Student’s *t*-test was used for statistical analysis. *** *p* < 0.001, **** *p* < 0.0001.

To identify the factor responsible for peroxisomal lipid dysregulation in *Kras^G12D^* CRC, we analyzed the expression levels of 94 peroxisomal genes listed in the peroxisome database [22] and performed multiple *t*-tests. Among the genes tested, the expression level of *Crat* was significantly increased (*p* ≤ 0.05), whereas the expression level of *Nudt7* was significantly decreased (*p* ≤ 0.05) in *Kras^G12D^* CRC cells (LS174T, SNU-407, and SNU-C2A) compared with *Kras^WT^* CRC cells (Caco2, HT29, and COLO-320DM) with different genetic backgrounds [23,24,25] (Figure S1). In human CRC tumors, the expression levels of *Nos2, Sod2, Acot2, Xdh, Acot1, Crat, Crot, Phyh, Sod1,* and *Hao1* were significantly increased (*p* ≤ 0.05), whereas the expression levels of *Nudt7, Acsl5, Abcd3, Acsl3, Far1, Pex12, Rhoc, Acsl4, Ehhadh, Ech1, Dnajc10, Acaa1, Agps, Acsf3, Trim37, Pex6,* and *Hmgcl* were significantly decreased (*p* ≤ 0.05) in *Kras^G12D^* CRC tumors (Figure S1B,C; including functional categories for these genes according to peroxisome database). Comparison of gene profiles showed that *Crat* was significantly increased whereas *Nudt7* was significantly decreased both in *Kras^G12D^* CRC cells and *Kras^G12D^* CRC patients tumors (Figure S1A,B). The involvement of the carnitine system including *Crat* is well-known. The carnitine system alters the metabolic plasticity and supplies an energetic and biosynthetic demand of cancer cells [26]. Higher carnitine O-acetyltransferase (CRAT) expression is also known to contribute to maintaining a high metabolic plasticity in cancers and suppressing the carnitine system [27]. However, the function of *Nudt7* in cancer has not been well studied. Analysis of GSE8671 (comparison between 32 prospectively collected adenomas and normal mucosa from the same individuals) [28] also showed a significant decrease in *Nudt7* in CRC (Figure 2A). We observed a drastic decrease in the level of *Nudt7* in *Kras^G12D^* CRC compared to *Kras^WT^* CRC tumors (Figure 2B).

### 2.2. Dysregulation of Lipid Metabolism by Nudt7 Suppression Is Involved in Development and Progression of Kras^G12D^ CRC 

**Figure 3:**
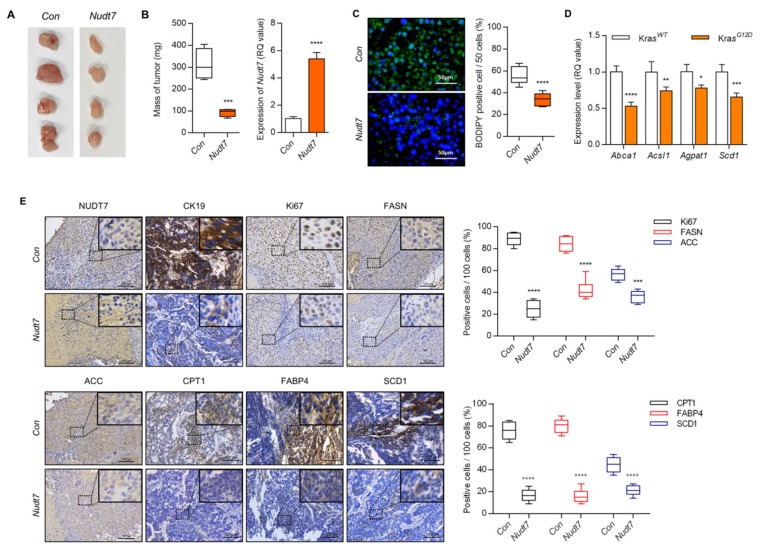
*Nudt7* is involved in the progression of *Kras^G12D^* CRC. (**A,B**) Cells transduced with lentivirus containing the control and *Nudt7* in LS174T cells were grafted into nude mice. Each tumor mass was measured and the expression level of *Nudt7* was confirmed by real-time PCR and is presented as the fold change compared with the control (Con). Results are representative of at least 4 independent experiments. (**C**) BODIPY staining in control and *Nudt7*-grafted tumors, and BODIPY-positive cells were counted for every 50 cells in 3 different fields. Results are representative of at least 3 independent experiments. Scale bars: 50 μm. (**D**) Expression level of lipid metabolic genes and presented as the fold change compared with Con. *Rn18s* was used as an endogenous control. Results are representative of at least 3 independent experiments. (**E**) Immunohistochemical staining with cytokeratin 19 (CK19), Ki67, FASN, ACC, CPT1, FABP4, and SCD1. CK19 was used as a marker for epithelial tissue. Positive cells were counted for every 100 cells in 3 different fields at 200× magnification. Results are representative of at least 3 independent experiments. The dotted line boxes were enlarged in the upper right corner of each image. Scale bars: 100 μm. Values are means + SD. An unpaired Student’s *t*-test was used for statistical analysis. * *p* ≤ 0.05, ** *p* < 0.01, *** *p* < 0.001, **** *p* < 0.0001.

To identify the functional role of *Nudt7* in lipid homeostasis, we analyzed lipid accumulation via modulation of *Nudt7*. Lipid accumulation and the number of BODIPY-positive cells (average of 0.2-fold with *Nudt7* overexpression (*Nudt7)*, 1.8-fold with *Nudt7* knockdown (*shNudt7*) compared to *Kras^G12D^* CRC cells), were significantly increased in *Kras^G12D^* CRC cell lines, SNU-407, and SNU-C2A, and this increase was dramatically reduced by overexpression of *Nudt7* (Figure S2). Knockdown of *Nudt7* into *Kras^WT^* Caco2 cells significantly increased the accumulation of medium chain fatty acids (MCFAs), LCFAs, and VLCFAs (Figure S3A). The increased expression levels of lipogenic genes such as *Acaca, Cd36, Cpt1b, Fasn, Ldlr, Pparg, Scd1, and Vldlr* by *shNudt7* into *Kras^G12D^* cells, SNU-407 and SNU-C2A (Figure S3B) were recovered by *Nudt7* restoration. Moreover, increased levels of *Abca1*, *Acsl1*, *Agpat1*, and *Scd1* by *Kras^G12D^* overexpression (*Kras^G12D^*) into *Kras^WT^* Caco2 cells (Figure S4A) were recovered by *Nudt7* restoration. In a genetically engineered mouse model of inducible CRC, *Villin-CreER^T2^, Apc^f/f^, Trp53^f/f^,* and *tetO-LSL-Kras^G12D^* [29], the expression levels of CPT1, FABP4, and SCD1 (Figure S4B) were significantly upregulated whereas the expression level of *Nudt7* was dramatically decreased when *Kras^G12D^* was turned on (+DOX; *Kras^ON^*; 0.51-fold of *Kras^OFF^*) (Figure 2C). The subcutaneous mouse model of *Kras^G12D^* cells, LS-174T, showed that overexpression of *Nudt7* significantly decreased tumor mass (3.36-fold of control (Con))(Figure 3A,B). The number of BODIPY-positive cells (0.61-fold of Con)(Figure 3C), the expression level of lipogenic genes such as *Abca1* (0.53-fold of Con), *Acsl1* (0.74-fold of Con), *Agpat1* (0.77-fold of Con), and *Scd1* (0.65-fold of *Con*)(Figure 3D), and the number of Ki67- (0.27-fold of Con), FASN- (fatty acid synthase; 0.5-fold of Con), ACC- (acetyl-CoA carboxylase α; 0-64 fold of Con), CPT- (0.2-fold of Con), FABP4- (0.2-fold of Con), and SCD1-positive cells (0.5-fold of Con)(Figure 3E) were also significantly decreased in xenograft tumors with *Nudt7* overexpression.

**Figure 4:**
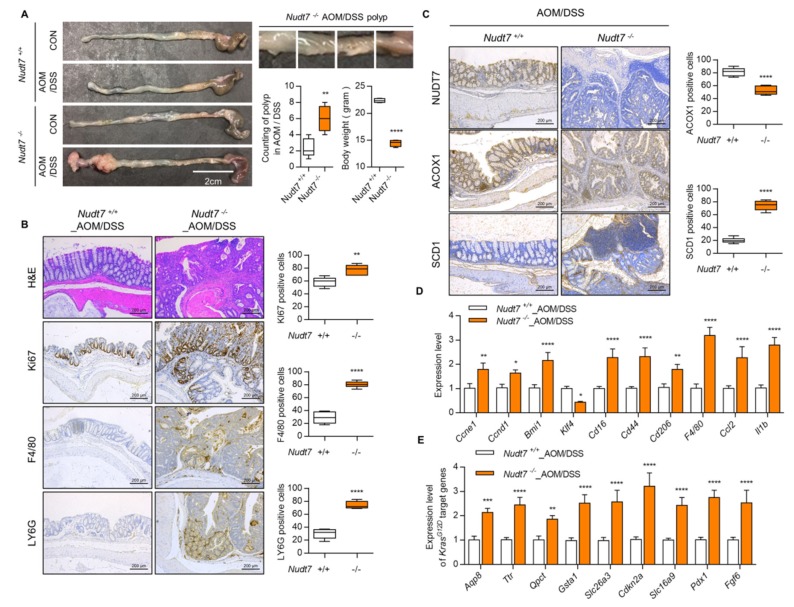
AOM/DSS (azoxymethane/dextran sulfate sodium)-treated *Nudt7^−/−^* mice increases the possibility of CRC development. (**A**) Mice were treated with AOM/DSS and polyp number was counted. Scale bars: 2 cm. (**B,C**) Immunohistochemistry with hematoxylin and eosin (H&E), Ki67, F4/80, LY6G, NUDT7, ACOX1, and SCD1 (*n* = 4) and positive cells were counted for every 100 cells in 3 different fields at 100× magnification in *Nudt7^+/+^* and *Nudt7^−/−^* colons treated with AOM/DSS. Results are representative of at least 3 independent experiments. Scale bars: 200 μm. (**D,E**) Transcriptional levels of proliferation and inflammatory marker genes and *Kras^G12D^* target genes in *Nudt7^+/+^* and *Nudt7^−/−^* colons treated with AOM/DSS. *Rn18s* was used as an endogenous control. Results are representative of at least 3 independent experiments. Values are means + SD. An unpaired Student’s *t*-test was used for statistical analysis. * *p* ≤ 0.01, ** *p* < 0.01, *** *p* < 0.01, **** *p* < 0.0001.

To identify the role of *Nudt7* in the development of CRC, we induced colonic polyp formation using an azoxymethane/dextran sulfate sodium (AOM/DSS) method using *Nudt7* knock-out (KO, *Nudt7^−/−^)* mice [30]. AOM/DSS challenge has been shown to induce acute inflammation in the colon with increased immune cell infiltration [31]. Lymphocyte antigen 6 complex locus G6D (LY6G), protein tyrosine phosphatase receptor type C (CD45), and adhesion G protein-coupled receptor E1 (F4/80) are used as inflammatory infiltration marker for colon cancer progression and metastasis marker [27,32,33]. *Nudt7^−/−^* mice developed more polyps (Figure 4A) and adenocarcinoma (Figure S5) and showed the dramatically increased Ki67- (expressed in colonic glands and proliferating cells; 1.32 fold of *Nudt7^−/−^*_AOM/DSS colon), F4/80- (expressed in immune cells; 2.79 fold of *Nudt7^−/−^*_AOM/DSS colon), LY6G- (expressed immune cells; 2.47 fold of *Nudt7^−/−^*_AOM/DSS colon), and SCD1- (3.70 fold of *Nudt7^−/−^*_AOM/DSS colon) positive cells in colons (Figure 4B,C). The increased levels of peroxisomal coenzyme A diphosphatase 7 (NUDT7) and acyl-CoA oxidase 1 (ACOX1) expressed in the epithelial cells and colonic glands in *Nudt7^+/+^*_AOM/DSS colon were significantly decreased in *Nudt7^−/−^*_AOM/DSS colon (Figure 4C). The expression level of genes involved in CRC progression [34] and target genes of *Kras^G12D^* mutation [35,36] (Figure 4D,E) were also significantly increased in colons of *Nudt7^−/−^* mice compared to the those of *Nudt7^+/+^* _AOM/DSS mice.

### 2.3. Increased Palmitic Acid Level by Nudt7 Suppression Is Responsible for CRC Development through Activation of Wnt/β-Catenin Signaling

**Figure 5:**
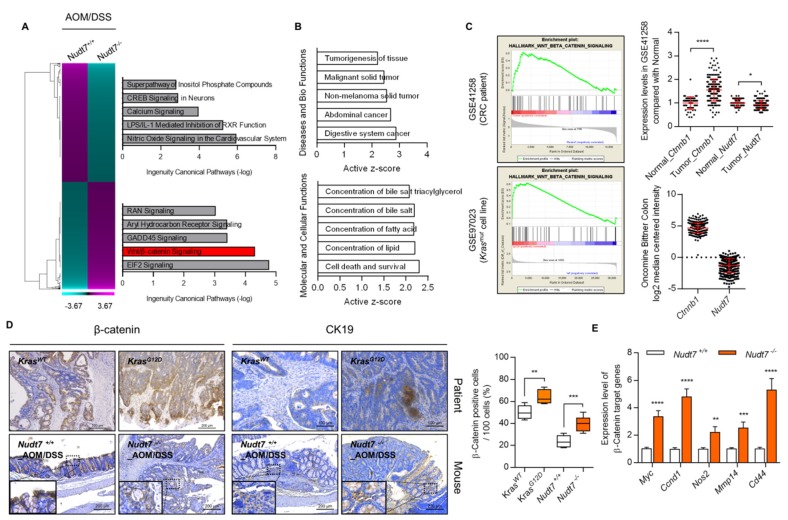
Suppression of *Nudt7* increases β-catenin signaling. (**A**) Canonical pathway was analyzed using IPA software (Qiagen, Redwood, CA, USA) from microarray data of *Nudt7^+/+^* and *Nudt7^−/−^* colons treated with AOM/DSS. (**B**) Active *z*-score of disease and biological functions or molecular and cellular functions were analyzed using IPA software. (**C**) GSEA analysis using GEO datasets (CRC patient biopsy dataset, GSE41258 and *Kras^G12D^* mutation CRC cell line dataset, GSE97023) and expression levels of *Nudt7* and *Ctnnb1*. (**D**) Immunohistochemical staining with β-catenin and CK19 and positive cell count in *Nudt7^+/+^* and *Nudt7^−/−^* mice treated with AOM/DSS (*n* = 4; 100× magnification; scale bars: 200 μm) and *Kras^G12D^*-derived CRC patient (*n* = 4; β-catenin, 100× magnification, scale bars: 200 μm; CK19, 200× magnification, scale bars: 100 μm) and positive cells were counted for every 100 cells in 3 different fields. CK19 was used as a marker for epithelial tissue. Results are representative of at least 3 independent experiments. The dotted line boxes were enlarged in the bottom left corner of each image. (**E**) Transcriptional levels of β-catenin target genes and presented as the fold change compared with *Nudt7^−/−^* mice. *Rn18s* was used as an endogenous control. Results are representative of at least 3 independent experiments. Values are means + SD. An unpaired Student’s *t*-test was used for statistical analysis. * *p* ≤ 0.05, ** *p < *0.01, *** *p* < 0.01, **** *p <* 0.0001.

**Figure 6:**
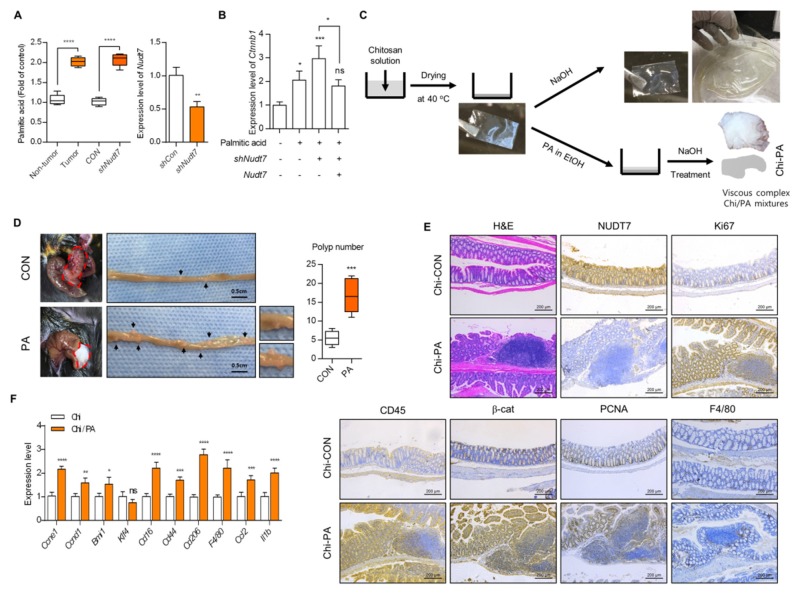
Accumulated palmitic acid is responsible for activation of β-catenin signaling. (**A**) Lipidomics was applied and the expression level of palmitic acid (PA) was analyzed in CRC tumors or *shNudt7* introduced *Kras^WT^* CRC cells (left panel) (*n* = 4). The efficiency of *shNudt7* was confirmed using *Kras^WT^* CRC cells (right panel). (**B**) *Kras^WT^* Caco2 cells were introduced with *Nudt7* or *shNudt7* in the presence of PA and the expression level of β-catenin was analyzed by real-time PCR and represented as the fold change compared with the negative control (-palmitic acid/-*shNudt7*/-*Nudt7)*. Results are representative of at least 3 independent experiments. *Rn18s* was used as an endogenous control. (**C**) Schematic diagram of generating Chi or Chi/PA film. (**D**) Chi or Chi/PA film was attached in the colon of *Nudt7^+/+^* mice for one week and polyp number was counted. Scale bars: 0.5 cm. (**E**) Immunohistochemistry with H&E, NUDT7, Ki67, CD45, β-catenin, PCNA, and F4/80 *(n* = 5) colon-attached Chi or Chi/PA film. Results are representative of at least 3 independent experiments and for every 100 cells in 3 different fields at 100× magnification. Scale bars: 200 μm. (**F**) Expression level of CRC-related genes in colon-attached Chi or Chi/PA film and represented as the fold change compared with colon-attached Chi film. Results are representative of at least 3 independent experiments. * *p* ≤ 0.05, ** *p < *0.01, *** *p* < 0.01, **** *p <* 0.0001.

Ingenuity pathway analysis (IPA), using microarray data of *Nudt7^+/+^* and *Nudt7^−/−^* colons treated with AOM/DSS, suggested the Wnt/β-catenin signaling pathway as one of the upregulated pathways (Figure 5A) in *Nudt7^−/−^* colons challenged with AOM/DSS. IPA indicated the increase in the concentration of lipids as an enriched molecular and cellular function in *Nudt7^−/−^* colons (Figure 5B). Significantly increased levels of *Ctnnb1* (186 tumor vs. 54 normal; 1.55-fold) and decreased levels of *Nudt7* (0.93-fold) were also observed in 7 *Kras^G12D^* cell lines (CL-40, Colo678, EB, IS1, IS3, LS174T, and TC71 cell line) compared with 12 *Kras^WT^* cell lines (Caco2, CL-34, Co115, Colo205, Colo320, HT29, KM12, NCI-H508, RKO, and SW48 cell line) and Bittner colons according to analysis of GSE41258 and Oncomine (Figure 5C). We found that the expression level of β-catenin was significantly increased both in *Nudt7^−/−^* mice colons treated with AOM/DSS and the patient colon of *Kras^G12D^* tumor (Figure 5D). The expression levels of β-catenin target genes, such as *Myc* (3.3-fold of *Nudt7^+/+^* mice colon), *Ccnd1* (4.8-fold of *Nudt7^+/+^* mice colon), *Nos2* (2.2-fold of *Nudt7^+/+^* mice colon), *Mmp14* (2.5-fold of *Nudt7^+/+^* mice colon), and *Cd44* (5.2-fold of *Nudt7^+/+^* mice colon), were also significantly increased in *Nudt7^−/−^* mice colons compared with *Nudt7^+/+^* mice colons (Figure 5E). Restoration of *Nudt7* by tail-vein injection into *Nudt7^−/−^* mice significantly reduced the development of adenoma observed in *Nudt7^−/−^* mice colons (Figure S6) and the number of FABP4-, FASN-, Ki67-, CD45-, and SCD1-positive cells (Figure S6). Analysis of fatty acid composition using clinical adjacent normal (non-tumor) and CRC tissues as well as Caco2 cells transfected with *shCon* or *shNudt7* showed a significant increase in palmitic acid (PA) (Figure 6A and Table S1), suggesting the possible involvement of *Nudt7* in the accumulation of PA. To investigate the involvement of PA in the activation of the Wnt/β-catenin signaling pathway induced by *Nudt7*, *Kras^WT^* Caco2 cells were treated with PA. Exposure of PA into *Kras^WT^* Caco2 cells significantly increased the transcription level of *Ctnnb1* (Figure 6B and Figure S7B), suggesting that increased levels of palmitic acid by *Nudt7* deficiency may be involved in the activation of the Wnt/β-catenin signaling pathway. 

For further verification of the PA role in CRC development, we produced the chitosan film (Chi) and chitosan/palmitate (Chi/PA) complex (Figure 6C) which was attached in the colon in *Nudt7^+/+^* mice for a week. We observed a significantly increased number of polyps (Figure 6D). We observed the increase in infiltration of inflammatory cells, a key feature of sporadic adenomatous colonic polyps with an increased number of Ki67-, CD45-, β-catenin-, proliferating cell nuclear antigen (PCNA)-, and F4/80-positive cells (Figure 6E) in the Chi/PA-attached *Nudt7^+/+^* colon polyp. The expression level of genes involved in CRC development and progression also increased in Chi/PA-attached *Nudt7^+/+^* colons (Figure 6F).

## 3. Discussion

Since lipid metabolism is known to be closely involved in various regulatory and developmental signaling pathways through regulation of cell growth and proliferation [37], it is not surprising that lipogenic regulatory pathways are dysregulated in cancer. Among the cellular organelles involved in lipid metabolism, our laboratory [38] previously suggested the importance of peroxisomes and their inter-connection with mitochondria in maintaining lipid homeostasis. Other studies also suggest that peroxisome plays a central role in cellular lipid metabolism, and particularly in fatty acid oxidation, which cannot be replaced by mitochondria [39,40]. β-oxidation of VLCFAs and branched chain fatty acids preferentially occurs in peroxisomes [41] and patients with peroxisomal disorders show increased serum levels of VLCFAs and branched chain fatty acids [42,43]. Here, in CRC patients, we found elevated levels of VLCFAs, suggesting the possible involvement of peroxisomal dysfunction in the pathogenesis of CRC. We observed high levels of peroxisomal lipid ROS and dysregulation of peroxisomal function in *Kras^G12D^* CRC cells compared with *Kras^WT^* CRC cells, suggesting that revealing and targeting the underlying mechanisms of peroxisomes in *Kras^G12D^* CRC could provide a possible strategy to develop effective therapeutic intervention against *Kras*-dependent malignancies. 

To identify the possible responsible genes in dysregulation of lipid homoeostasis in *Kras^G12D^* CRC, transcriptomic analysis identified *Nudt7*, a peroxisomal gene. NUDT7 is known to mediate the cleavage of CoA, CoA ester, oxidized CoA to 3, 5-adenosine diphosphate (ADP) and 4-phosphopantetheine, and regulates CoA and acyl CoA levels in peroxisome [44,45]. Since the important role of CoA homeostasis in tumor growth and malignant progression has been suggested [46], NUDT7 may play an important role in cancer development by altering CoA pools. However, the exact function and role of NUDT7 has not been well studied. Here, we found that downregulation of *Nudt7* leads to VLCFA accumulation, and overexpression of *Nudt7* inhibits in vitro proliferation and in vivo xenograft tumor growth of *Kras^G12D^* CRC cells. AOM/DSS-challenged *Nudt7^−/−^* colon exhibited significant increases in the number of CD45-, F4/80-, and LY6G-positive cells suggesting *Nudt7* deficiency may be attributed to the induced number of infiltration immune cells in the colon of AOM/DSS-treated mice. *Nudt7* is important in the recruitment of neutrophil and monocytes/macrophages to the inflammatory sites following AOM/DSS challenge and may contribute to the induction of inflammatory cytokines, in line with our findings that show that *Nudt7* deficiency stimulates inflammatory responses in mice and humans with osteoarthritis [30]. Moreover, *Nudt7^−/−^* mice showed a significantly more pronounced increase in the expression levels of genes involved in the development of CRC, suggesting NUDT7 as a novel potent tumor suppressor for CRC.

Aberrant Wnt signaling is associated with many types of cancers [47,48] suggesting Wnt signaling is an attractive and effective target for therapeutic strategy. Activation of Wnt signaling is frequently observed in human CRC patients [49,50]. Activation of Wnt signaling acts as a key oncogenic driver [32], which is crucial for stem cell maintenance in CRC [51]. The *Adenomatous polyposis coli* (*Apc*) tumor suppressor gene interacts with β-catenin [52] and loss of function of *Apc* results in overactive T-cell factor (*TCF*) 4/β-catenin signaling [53]. Other studies reported the crosstalk between Wnt/β-catenin and Ras-Erk pathways in the colorectal tumorigenesis [54,55]. Wnt signaling is involved in the stability of Ras through regulation of glycogen synthase kinas 3β-mediated Ras phosphorylation [56]. In hepatocellular carcinoma, Wnt signaling modulates lipid homeostasis in a Ras-dependent manner [57]. In this study, we found that Ras activity modulates the expression level of NUDT7 in an opposite manner, and decreased levels of NUDT7 deregulate lipid homeostasis by the upregulation of Wnt signaling. 

Fatty acids can influence cell and tissue metabolism and function through the modulation of immunity, inflammation, and cell signaling [34,35]. Fatty acids are associated with disease risk and contribute to the development of pathological conditions [58,59]. The important role of eicosanoids generated from cyclooxygenase and the lipoxygenase metabolism of arachidonic acid, an n-6 polyunsaturated fatty acid (PUFA), has been suggested in the development of CRC [60]. Here, we found that suppression of *Nudt7* induced the accumulation of palmitic acid and this accumulated palmitic acid acted as a driver of Wnt signaling. In several cancers including melanoma, breast cancer, and prostate cancer, cancer cells can uptake, incorporate, and use the exogenous palmitic acid to fuel their pathogenicity [61]. Palmitic acid triggers a non-canonical autophagic response in human cancer cells [62,63]. Palmitic acid increases CRC growth via the modulation of β-adrenergic receptor expression [64] or by modulation of Wnt signaling [65]. Several studies proposed the connection of palmitic acid to cancer promotion/suppression via Wnt/β-catenin signaling [66]. However, the responsible regulatory factor induced by palmitic acid has not been well studied. As a possible regulatory factor, we found that palmitic acid altered the expression level of microRNA (miR)-32-5p and increased the level of miR-32-5p, stimulating Wnt/β-catenin signaling (Figure S7). Introduction of PA into *Kras^WT^* Caco2 cells increased the expression level of miR-32-5p known as onco-miRs (Figure S7A) and introduction of miR-32-5p increased expression level of β-catenin (Figure S7B). Furthermore, we observed an increased level of miR-32-5p in CRC patient tumors and *Nudt7^−/−^* mice colons (Figure S7C). Several reports [67,68,69] suggested that PA is involved in tumorigenesis by modulating the level of miRs. PA increases the invasiveness of pancreatic cancer cells by decreasing the level of miR-29c [70]. However, the exact function and mechanism of miR-32-5p in CRC induced by suppression of *Nudt7* is currently under study in our laboratory. 

In summary, our data indicate that suppression of *Nudt7* may be responsible for tumorigenic activity of *Kras^G12D^* CRC through upregulation of Wnt/β-catenin signaling and accumulation of palmitic acid. Restoration of *Nudt7* expression may be developed as a therapeutic strategy to treat patients with CRC, especially cases with *Kras^G12D^* mutation. 

## 4. Materials and Methods

### 4.1. Clinical Samples

Tumor specimens were collected from colorectal cancer patients who underwent resection of colorectal tumors at Wonkwang University Hospital (Iksan, Korea). Sample collection was approved by the Human Subjects Committee of Wonkwang University (Chunbuk, Korea, #WKUH 201401-BR-003).

### 4.2. Ethics Approval

All the animal experiments were conducted according to the protocol approved by the Animal Care and Use Committee of Wonkwang University (#WKU-18-22). Human colon tissue collection was approved by the Human Subjects Committee of Wonkwang University Hospital and studies were performed in compliance with the institutional guidelines (#WKUH 201401-BR-003). Written informed consent was obtained from all adult patients or at least one guardian of each patient prior to the start of the experiment.

### 4.3. Animals

The AOM/DSS mouse model [71] was used to examine the mechanisms of human colorectal carcinogenesis. Eight-week-old C57BL/6 wild mice and *Nudt7^−/−^* mice [30] were injected intraperitoneally with azoxymethane (AOM; 12.5 mg/kg, Sigma-Aldrich, Louis, MO, USA). We used three cycles of feeding mice with 2% dextran sulfate sodium (DSS) in the drinking water for 5 days, which was replaced on day 6 with autoclaved drinking water without DSS for 5 days. After 30 days, mice were sacrificed, and colon tissues were collected. *Nudt7* were cloned into pLenti-CMV-RFP-puro (Abm, Richmond, BC, Canada) lentiviral vector and introduced into the mice by tail-vein injection of *Nudt7*. Tissue sections (formalin-fixed, paraffin-embedded) of mouse strain *Villin-CreER^T2^; Apc^f/f^; Trp53^f/f^; tetO-LSL-Kras^G12D^* were generous gifts from Drs. DePinho and Boutin [29]. 

### 4.4. Mouse Xenograft Model

Cancer cells (5 × 10^5^ cells/100 μL/site) were suspended in Matrigel Matrix (Corning, Corning, NY, USA), then subcutaneously injected into the flank of 6-week-old male nude mice (BALB/c-nude). Injected tumor size was measured every day with calipers. Four weeks after injection, tumors were excised from euthanized mice and the tumor mass and weight were measured.

### 4.5. Cell Culture

Six human colon cancer cell lines with documented *Kras G12D* mutations (*Kras^G12D^*), LS-174T, SNU-407, and SNU-C2A, and three *Kras* wild-type (*Kras^WT^*) colon cancer cells, Caco2, COLO-320DM, and HT29, were purchased from American Type Culture Collection (ATCC, Manassas, VA. USA) or Korea Cell Line Bank (KCLB, Seoul, Korea). Enriched cells were then cultured at sub-clonal density (1–10 cells/cm^2^) with complete growth medium of each cell line at 37 °C and 5% CO_2_ atmosphere. Caco2 was cultured in MEM medium (Gibco-Invitrogen, Waltham, MA, USA) supplemented with 20% FBS (Gibco-Invitrogen) at 37 °C and 5% CO_2_; LS-174T, SNU-407, SNU-C2A, HT-29, and COLO-320DM were cultured in RPMI 1640 medium (Gibco-Invitrogen) with 10% FBS. For the palmitate treatments, cells were treated with 50 μM conjugated palmitate (Sigma-Aldrich) with 10% (w/v) fatty acid free BSA (Sigma-Aldrich) in the DMEM medium. Cell lines used in silico analysis, in vitro, and in vivo experiments, as listed in Table S2.

### 4.6. Plasmids and Lentiviral Packaging 

*Nudt7* expression vector was generated by cloning a *Nudt7* coding sequence (CDS) in the pLenti-CMV-RFP-puro (Abm) lentiviral vector. *Nudt7*-specific shRNA was cloned in the pLKO.1-TRC cloning vector (Addgene, Watertown, MA, USA). Empty vector was used as a control. Lentivirus was packaged in HEK293T cells using a Lentifectin (Abm) and concentrated using Lenti-X Concentrator (Clonetech, Mountain View, CA, USA). 

### 4.7. Real-Time PCR

The real-time PCR was performed with RealMIX SYBR Kit, Hi-Rox (Geneer, Daejeon, Korea) in a Step One Plus Real-Time PCR System (Applied Biosystems, Beverly, MA, USA). The reaction mixtures were incubated at 95 °C for 10 min followed by 40 cycles at 95 °C for 10 s, 57 °C for 10 s, and 72 °C for 15 s. *18S ribosomal RNA* (*Rn18s)* or *actin beta* (*Actb)* was used as the housekeeping gene. The primer sequence used in this study is listed in Table S3. The fold change in the target genes was calculated based on 2^ΔΔCt^ method [72].

### 4.8. Quantification of miRNA

For miRNA quantification analysis, the cDNA samples were synthesized using a Mir-X miRNA First Strand Synthesis Kit (Clonetech). Quantitative RT-PCR was performed using Mir-X qRT-PCR SYBR Kit (Clontech) and miR-32-5p-specific forward primer (Genolution) in a Step One Plus Real-Time PCR System. Quantitative RT-PCR conditions were used per the manufacturer’s protocols. The expression of miR-32-5p was normalized with U6 small nuclear RNA (endogenous control; Qiagen, Redwood, CA, USA).

### 4.9. Peroxisomal Gene Profiling

RNA isolated using RNAiso (TaKaRa, Shiga, Japan) was reverse-transcribed using the AccuRT Genomic DNA Removal Kit (Abm) and oligo dT primers according to the manufacturer’s protocol. The relative abundance of each gene was determined using RT-PCR with specific primers for 94 peroxisomal genes. *Actb* and *Rn18s* were used as the endogenous control genes. Gene expression data were analyzed and visualized using PermutMatrix software (version# 1.9.3). The primer sequence used in this study is listed in Table S3.

### 4.10. Microarray Using RNA Isolated from Paraffin Section

Total RNA was extracted using a RecoverAllTotal Nucleic Acid Isolation kit (ThermoFisher Scientific, Waltham, MA, USA) from FFPE (formalin-fixed paraffin-embedded) CRC samples of *Nudt7*^+/+^ and *Nudt7^−/−^* mice treated with AOM/DSS. mRNA expression analysis was performed with the Affymetrix GeneChip Whole Transcript PLUS reagent method. cDNA was synthesized using a GeneChip Whole Transcript Amplification Kit. Fragmented sense cDNA was biotin-labeled with TdT (terminal deoxynucleotidyl transferase) using the GeneChip WT Terminal Labeling Kit, and hybridized to the Affymetrix GeneChip Mouse 2.0 ST Array for 16 h at 45 °C. The hybridized array was washed and stained on a GeneChip Fluidics Station 450 and scanned on a GCS3000 Scanner (Affymetrix, Waltham, MA, USA). Signal values were computed using the Affymetrix GeneChip Command Console software. 

### 4.11. Lipid Accumulation and ROS Staining

To measure lipid accumulation, cells were fixed with 4% paraformaldehyde for 10 min. After washing ice-cold 1× PBS, cells were stained with BODIPY or HCS LipidTOX Deep Red neutral lipid stain (ThermoFisher Scientific) for 10 min at room temperature. After PBS washing, cells were stained with DAPI (4’,6-diamidino-2-phenylindole) that was diluted in PBS. Stained cells were visualized and captured with the EVOS FL Auto Cell Imaging System (ThermoFisher Scientific).

### 4.12. Immunohistochemistry

Colon tissues were fixed in 10% neutral buffered formalin and paraffin-embedded after the dehydrate process. Paraffin-embedded tissue blocks were sliced into 5 μm sections for hematoxylin and eosin (H&E) staining and immunohistochemistry. The sections were deparaffinized, rehydrated, and antigen retrieval was performed using a pressure cooker with 0.01 M citrate buffer at pH 6.0. Intrinsic peroxidase activity was blocked using 1% hydrogen peroxide, and 10% normal goat serum (NGS; Vector Laboratories, Burlingame, CA, USA) was used for 1 h to block. The slides were then incubated overnight at 4 °C. The following antibodies were used for immunohistochemistry overnight at 4 °C (1:200 dilution): ACC (acetyl-CoA carboxylase, Abcam, Cambridge, MA, USA), ACOX1 (acyl-CoA oxidase 1, Abcam), β-catenin (BD Biosciences, San Jose, CA, USA), Ki67 (proliferation marker protein ki-67, Abcam), CD45 (leukocyte common antigen, Abcam), CK19 (cytokeratin 19, epithelial marker, Abcam), CPT1 (carnitine palmitoyltransferase 1, Abcam), FABP4 (fatty acid binding protein, Abcam), FASN (fatty acid synthetase, Abcam), F4/80 (staining for macrophage, ThermoFisher Scientific), LY6G (lymphocyte antigen 6 complex locus G6D, staining for monocytes, granulocytes and neutrophils, ThermoFisher Scientific), PCNA (proliferating cell nuclear antigen, Abcam), and SCD1 (stearoyl-CoA desaturase, Abcam). For mouse NUDT7 immunohistological detection, NUDT7 C-terminal specific peptide (6–20 amino acids, GLPEPVRNNLIDDAK-C) was synthesized by Ab frontier (Seoul, Korea). After incubation with the horseradish peroxidase (HRP)-conjugated secondary antibody (Rabbit or mouse IgG-heavy and light chain antibody, 1:500 dilutions; Bethyl Laboratories, Montgomery, TX, USA) for 1 h at room temperature, antigen signals were detected using the ImmPACT^®^ DAB Peroxidase (HRP) Substrate Kit (Vector Laboratories). All sections were counterstained with hematoxylin. Stained cells were visualized and captured with the Leica Imaging System (Wetzlar, Germany). Positive staining integrity was measured using IHC Profiler of Image J software (version 1.52a, NIH, Bethesda, MD, USA) [73,74].

### 4.13. Western Blot Analysis

Nuclear and cytoplasmic proteins were prepared using Nuclear/Cytosol Fractionation Kit (BioVision, Milpitas, CA, USA) and concentration was measured using the Pierce BCA Protein Assay Kit (Pierce, Waltham, MA, USA). We electrophoresed 40 μg protein on SDS-PAGE gel and transferred to a nitrocellulose membrane (GE Healthcare Life Sciences, Pittsburgh, PA, USA). Membranes were blocked with 5% skim milk in TBS-0.1% Tween 20 (TBS-T) overnight at 4 °C and immunoblotted with the following antibodies (1:1000 dilution): β-catenin (Abcam), GAPDH (Bioworld Technology, Bloomington, MN, USA), and Histone-H3 (Cell Signaling Technology, Danvers, MA, USA) for 1 h at room temperature. Then, membranes were incubated with the HRP-conjugated secondary antibody (1:2500 dilution) (α-Mouse, Enzo Lifesciences, Farmingdale, NY, USA; α-Rabbit, Enzo Lifesciences, Farmingdale, NY, USA) and the immunoreactive proteins were visualized with SuperSignal West Pico PLUS Chemiluminescent Substrate (ThermoFisher Scientific).

### 4.14. Preparation of Chitosan Films

Chitosan films were prepared using a solution casting method. Briefly, chitosan (100 mg) was hydrated in 5 mL of 5 N HCl solution, and 45 mL of distilled and deionized water (DDW) were added to the chitosan solution. After its complete dissolution, the chitosan was purified by dialysis (MWCO = 12–14 kDa, Spectra Por, Spectrum Chemical Mfg. Gardena, CA, USA) against DDW for 2 days. The purified chitosan solution was poured onto the Petri dishes (diameter: 35 mm, thickness: 10 mm) for solution casting. The chitosan films were fully dried at 40 °C for one day and maintained in a moisture-free desiccator until use. When we performed the experiments, the chitosan films were treated with sodium hydroxide (NaOH) for 10 min and then were washed with 10 mM PBS solutions several times.

### 4.15. Preparation of Chitosan/Palmitic Acid Composite Films (Chi/PA films)

To fabricate the Chi/PA films, the chitosan films were firstly prepared as described above. The mixture solutions of chitosan in water and palmitic acid in ethanol were poured onto the chitosan films. The solutions on the chitosan films were dried at 40 °C for one day and were maintained in a moisture-free desiccator until use. When Chi/PA films were treated with NaOH, the rigid structures of films were destroyed and instantaneously transitioned into a hydrogel-like state.

### 4.16. Gas Chromatograph/Mass spectrometry (GC/MS) Spectrometry

Total lipids were extracted using a chloroform/methanol (2:1 v/v) mixture. The extracted lipids were separated on a Sep-Pak Silica Cartilage column (Waters, Milford, MA, USA) and were transmethylated with 0.5 M CH_3_ONa in methanol by heating in a sealed tube at 70 °C for 1 h under nitrogen. The fatty acid methyl esters were extracted with hexane. Subsequent GC/MS analysis was performed according to the standard protocol [75].

### 4.17. Statistical Analysis

Statistical comparisons were performed on GraphPad Prism software (Version 6.0, San Diego, CA, USA) using unpaired *t*-test, two-way ANOVA, and multiple *t*-test for multiple variable experiments.

## 5. Conclusions

Our data indicate that suppression of *Nudt7* may be responsible for tumorigenic activity of *Kras^G12D^* CRC through upregulation of Wnt/β-catenin signaling and accumulation of palmitic acid. Restoration of *Nudt7* expression could be developed as a therapeutic strategy to treat patients with CRC, especially the cases with *Kras^G12D^* mutation.

## Supplementary Materials

The following are available online at https://www.mdpi.com/2072-6694/12/3/576/s1, Figure S1: Decreased level of *Nudt7* expression in *Kras^G12D^* tumor, Figure S2: *Nudt7* is involved in lipid homeostasis, Figure S3: Dysregulation of lipid homeostasis is induced by *Nudt7* deficiency, Figure S4: Dysregulation of lipid metabolism in *Kras^G12D^* CRC, Figure S5: Development of adenocarcinoma in *Nudt7^−/−^*_AOM/DSS colon, Figure S6: Restoration of *Nudt7* into *Nudt7^−/−^* mice reduces the expression levels of lipid metabolic and proliferation factors, Figure S7: Palmitic acid induced expression level of miR-32-5p and an increased level of miR-32-5p activates Wnt/β-catenin signaling, Table S1: Lipidomics analysis of CRC using gas chromatography/mass spectrometry (GC/MS), Table S2: List of cell lines used in this study, Table S3: List of real-time PCR primer used in this study.

## Abbreviations

ABCA1	ATP binding cassette subfamily A member 1	ACC	Acetyl-CoA carboxylase α
ACSL1	Acyl-CoA synthetase long chain fatty acid member 1	AGPAT	1-acyl-glycerol-3-phosphate acyltransferase
AOM	Azoxymethane	CD36	Fatty acid translocase
CD45	Protein tyrosine phosphatase receptor type C	CPT1	Carnitine palmitoyltransferase
CRC	Colorectal cancer	CK19	Cytokeratin 19
DSS	Dextran sulfate	F4/80	Adhesion G protein-coupled receptor E1
FABP4	Fatty acid binding protein 4	FASN	Fatty acid synthase
Ki67	Proliferation marker protein ki-67	KRAS	Kirsten rat sarcoma viral oncogene homolog
LCFA	Long chain fatty acid	LDLR	Low density lipoprotein receptor
LY6G	Lymphocyte antigen 6 complex locus G6D	MUFA	Mono-unsaturated fatty acid
NUDT7	Peroxisomal coenzyme A diphosphatase NUDT7	PPARG	Peroxisome proliferator activated receptor gamma
SCD1	Stearoyl-CoA desaturase-1	SFA	Saturated fatty acid
VLCFA	Very long chain fatty acid	VLDLR	Very low density lipoprotein receptor
