# Supplementary material for: NUDT7 Loss Promotes KrasG12D CRC Development
Source: Cancers (Basel). 2020 Mar 2;12(3):576. doi: 10.3390/cancers12030576 (PMC7139971; doi:10.3390/cancers12030576)
Supplement: Supplementary file 1 [file cancers-12-00576-s001.pdf]

Supplementary materials

A

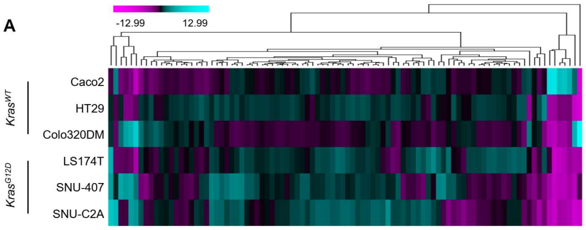

qRT-PCR analysis							
ΔΔCt	Caco2	HT29	COLO-320DM	LS174T	SNU-407	SNU-C2A	Multiple t-test
Crat	0.107	-0.359	0.252	-2.168	-3.223	-5.114	0.016
Nudt7	-0.585	-0.011	0.597	3.941	2.566	2.278	0.009

Genetic information						
Cell line	Caco2	HT29	COLO-320DM	LS174T	SNU-407	SNU-C2A
Disease	Carcinoma	Adeno-carcinoma	Adeno-carcinoma	Adeno-carcinoma	Adeno-carcinoma	Adeno-carcinoma
MSI status	MSS	MSS	MSS	MSI	MSI	MSI
HRAS	wt	wt	wt	wt	wt	wt
NRAS	wt	wt	wt	wt	wt	wt
KRAS	wt	wt	wt	G12D	G12D	G12D
BRAF	wt	p.V600E	wt	wt	wt	wt
PIK3CA	wt	wt	wt	H1047R	H1047R	WT
PTEN	positive	positive	positive	positive	positive	positive
TP53	E204X	R273H	R248W	wt	wt	wt

B

Kras	Age	Sex	Stage	Type
KrasWT_1	84	F	3BT3N1M0	Adenocarcinoma
KrasWT_2	57	M	2AT3N0M0	Adenocarcinoma
KrasWT_3	77	M	3BT3N2aM0	Adenocarcinoma
KrasWT_4	63	M	4AT3N2aM1a	Adenocarcinoma
KrasG12D_1	57	M	2AT3NoMo	Adenocarcinoma
KrasG12D_2	76	M	4BT3N2bM1b	Adenocarcinoma
KrasG12D_3	73	M	3CT3N2bM0	Adenocarcinoma
KrasG12D_4	76	F	2AT3N0Mo	Adenocarcinoma

C

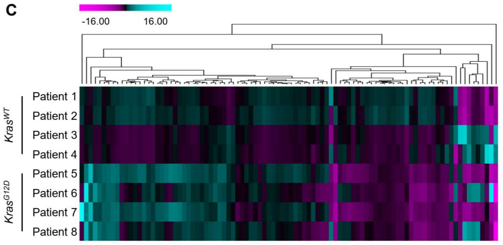

ΔΔCt	Wt_1	Wt_2	Wt_3	Wt_4	Mu_1	Mu_2	Mu_3	Mu_4	Multiple t-test
NOS2	-4.23	-3.83	5.15	2.92	-4.99	-12.29	-4.71	-12.35	0.0367
SOD2	-0.11	0.87	-0.44	-0.32	-4.36	-2.79	-4.25	-2.81	0.0005
ACOT2	0.42	0.74	-0.64	-0.52	-1.87	-2.50	-1.59	-2.40	0.0021
XDH	0.23	-0.09	-0.62	0.48	-1.92	-1.95	-2.35	-1.28	0.0012
ACOT1	0.54	0.60	-0.65	-0.49	-1.55	-2.22	-1.27	-2.15	0.0043
CRAT	-0.38	-0.21	0.14	0.45	-1.34	-1.89	-1.54	-2.14	0.0005
CROT	0.54	0.50	-1.01	-0.04	-1.09	-1.40	-1.24	-1.59	0.0124
PHYH	-0.22	0.07	-0.17	0.32	-1.19	-0.86	-1.08	-1.19	0.0003
SOD1	0.32	0.42	-0.45	-0.29	-0.84	-1.32	-0.75	-1.28	0.0072
HAO1	0.17	-0.29	0.09	0.03	-1.47	-0.45	-1.39	-0.78	0.0084
HMGCL	-0.01	0.14	-0.24	0.10	1.56	0.40	1.55	0.39	0.0300
PEX6	-0.39	-0.18	0.09	0.48	0.78	1.59	0.48	1.19	0.0165
TRIM37	0.11	0.27	-0.36	-0.02	1.12	1.15	1.06	1.03	0.0002
ACSF3	-0.42	-0.33	0.26	0.48	1.34	0.99	1.40	0.80	0.0050
AGPS	0.08	0.09	-0.18	0.01	1.44	0.79	1.50	0.86	0.0011
ACAA1	0.56	0.60	-0.74	-0.42	1.67	0.76	1.66	0.63	0.0371
DNAJC10	-0.45	-0.22	0.26	0.41	1.42	1.18	1.50	1.25	0.0008
ECH1	-0.02	0.14	-0.21	0.08	1.99	0.65	2.33	0.51	0.0263
EHHADH	-0.37	-0.22	0.23	0.37	2.21	0.46	2.64	0.56	0.0469
ACSL4	0.24	0.41	-0.44	-0.21	1.08	2.01	0.87	1.93	0.0057
RHOC	-0.24	-0.14	0.03	0.36	1.58	1.46	1.63	1.51	0.0000
PEX12	0.29	0.54	-0.51	-0.31	1.90	1.42	2.05	1.52	0.0010
FAR1	-0.09	0.13	-0.31	0.27	1.68	1.94	1.56	1.85	0.0000
ACSL3	0.25	0.15	-0.31	-0.09	2.10	1.85	2.11	1.97	0.0000
ABCD3	0.15	0.34	-0.39	-0.11	0.89	3.84	1.03	3.69	0.0287
ACSL5	-0.42	-0.23	0.15	0.50	5.58	1.28	5.58	1.27	0.0346
NUDT7	-0.04	-0.26	0.11	0.20	2.17	10.50	2.31	5.96	0.0374

Expression	Gene	Functional Categories (http://www.peroxisomedb.org)	Reference for cancers
Up-regulated	*Nos2*	Anti-inflammatory/ anti-microbial	[71-76]
	Sod1, Sod2	Superoxide dismutase	[77-81]
	Acot1, Acot2	Regulation of acyl-CoA/CoA ratio	[82]
	Xdh	Purines and pyrimidines	[83]
	Phyh	Alpha-oxidation	-
Down-regulated	*Acsf3, Acsl3, Acsl4, Acsl5*	Long/very long chain fatty acids activation,	[84-86]
	Agps, Far1	Etherlipid and plasmalogens synthesis	[87]
	Dnajc10	Peroxisome organization	-
	Abcd3	ABC transporters	[88-89]
	Pex12	ZN RING proteins	-
	Ehhadh	Long-chain dicarboxylic acids oxidation	[90]
	Ech1	Unsaturated fatty acid beta-oxidation	[91]
	Pex6	Peroxisomal AAA-ATPases	-
	Hmgcl	Lipid metabolism	[92]

Figure S1. Decreased level of *Nudt7* expression in $Kras^{G12D}$ tumor. (**A**) The expression level of peroxisomal genes in $Kras^{G12D}$ cell lines (LS-174T, SNU-C2A, and SNU-407) compared with $Kras^{WT}$ cell lines (Caco2, HT-29, COLO-320DM). (**B**) Characteristics of the tumor patients. (**C**) The expression level of Peroxisomal genes in CRC tumor biopsy with $Kras^{G12D}$ ($n = 4$) compared to CRC tumor biopsy with $Kras^{WT}$ ($n = 4$). Multiple t-test was used for statistical analysis (left panel), and categorized peroxisomal genes and references in various cancers include the colorectal cancer (right panel) [1–23].

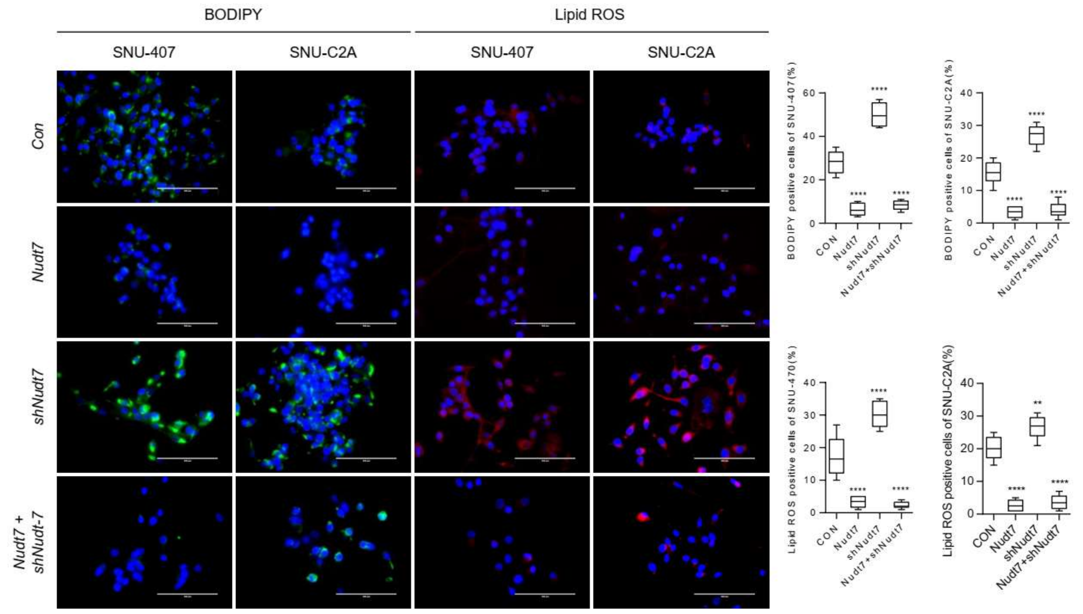

Figure S2. *Nudt7* is involved in lipid homeostasis. Lipid accumulation and lipid oxidative stress (ROS) were measured using BODIPY$^{493/503}$ and lipid deep red neutral lipid stain, respectively, and positive cells were counted. Scale bars: 100 µm.Values are means + SD. A two-tailed Student's *t*-test was used for statistical analysis. ** *P* < 0.01, **** *P* < 0.0001.

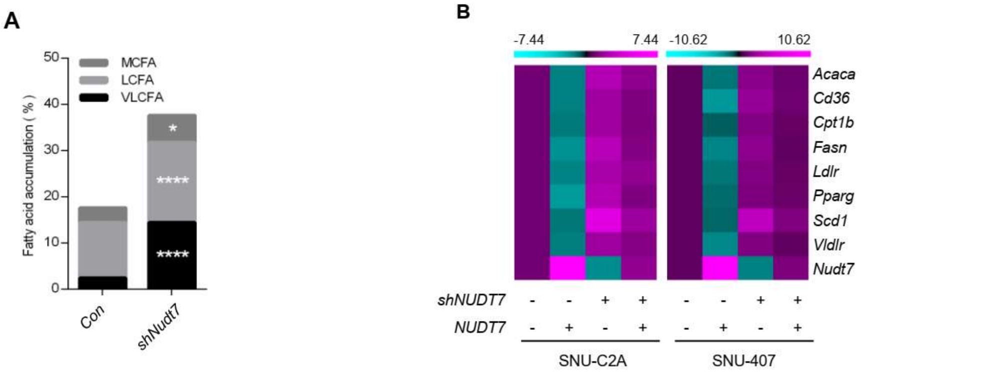

SNU-C2A				SNU-407				Gene
1.00	-1.24	2.47	1.49	1.00	-1.52	2.02	1.27	*Acaca*
1.00	-1.23	1.90	1.21	1.00	-2.46	2.45	1.35	*Cd36*
1.00	-1.14	1.95	1.17	1.00	-1.04	1.84	1.16	*Cpt1b*
1.00	-1.62	2.62	1.31	1.00	-1.95	2.13	1.04	*Fasn*
1.00	-1.34	2.13	1.60	1.00	-1.56	1.86	1.16	*Ldlr*
1.00	-1.84	2.44	1.21	1.00	-1.27	1.66	1.18	*Pparg*
1.00	-1.08	4.36	1.86	1.00	-1.14	3.98	1.78	*Scd1*
1.00	-1.20	1.87	1.36	1.00	-2.01	1.75	1.07	*Vldlr*
1.00	7.44	-1.51	1.60	1.00	10.62	-1.81	1.64	*Nudt7*
-	-	+	+	-	-	+	+	*shNudt7*
-	+	-	-	-	+	-	+	*Nudt7*

Figure S3. Dysregulation of lipid homeostasis is induced by *Nudt7* deficiency. (**A**) Lipidomics analysis of Caco2 cells transduced with lentiviruses containing shRNA specific to *Nudt7* (*shNudt7*) using gas chromatography/mass spectrometry (GC/MS; *n* = 3). (**B**) Expression level of genes involved in lipogenesis was analyzed by real-time PCR and normalized with *Rn18s* (*n* = 3).

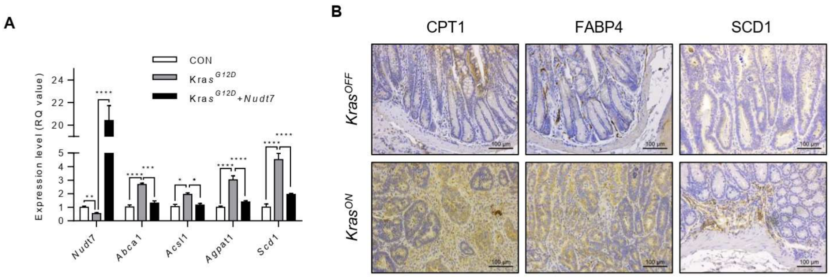

Figure S4. Dysregulation of lipid metabolism in *Kras^{G12D}* CRC. (**A**) Expression level of *Nudt7* and lipid metabolic genes in Caco2 cells transfected with *Kras^{G12D}* in the absence or presence of *Nudt7*. *Rn18s* was used as an endogenous control (n = 3). (**B**) Immunohistochemistry with CPT1, FABP4, and SCD1 using tamoxifen-inducible *Villin-CreER^{T2}; Apc$^{f/f}$; Trp53$^{f/f}$; tetO-LSL-Kras^{G12D}* mice (+Dox) (n = 5). Scale bars: 100 μm.

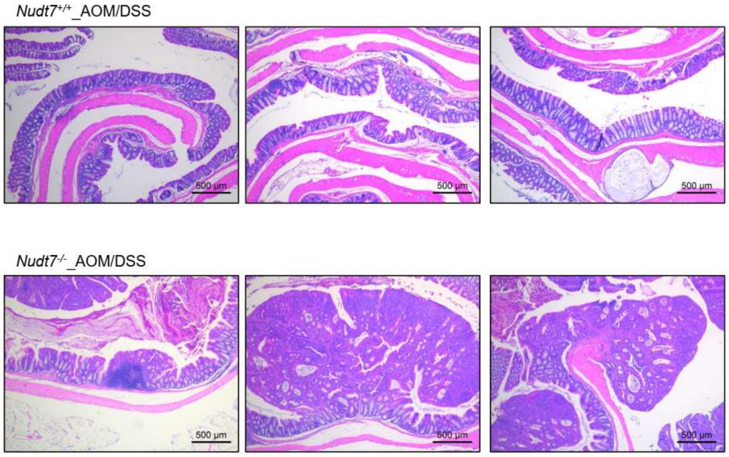

Figure S5. Development of adenocarcinoma in *Nudt7$^{-/-}$*_AOM/DSS colon. Low magnified H&E images (40× magnification, Scale bars: 500 μm) for Figure 4B.

Figure S6

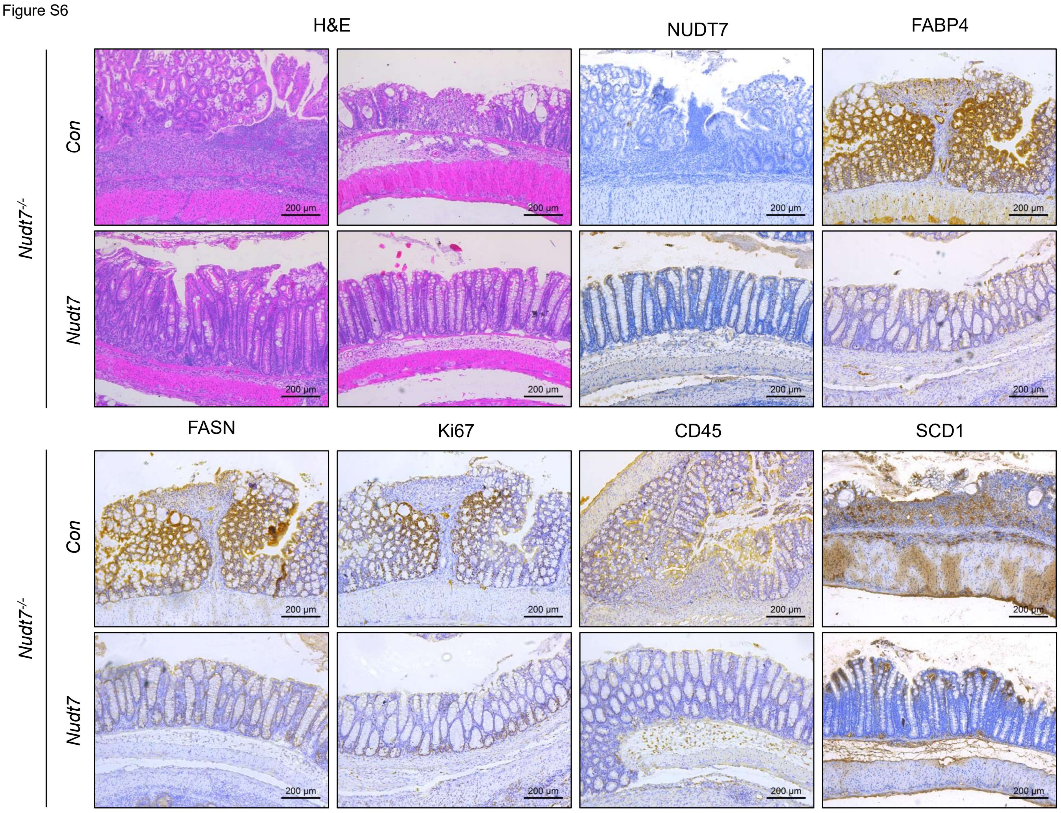

Figure S6. Restoration of *Nudt7* into *Nudt7*$^{-/-}$ mice reduces the expression levels of lipid metabolic and proliferation factors. Lentiviruses containing *Nudt7* were injected into tail vein of *Nudt7*$^{-/-}$ mice and stained with NUDT7, FABP4, FASN, Ki67, CD45, and SCD1. Scale bars: 100 µm.

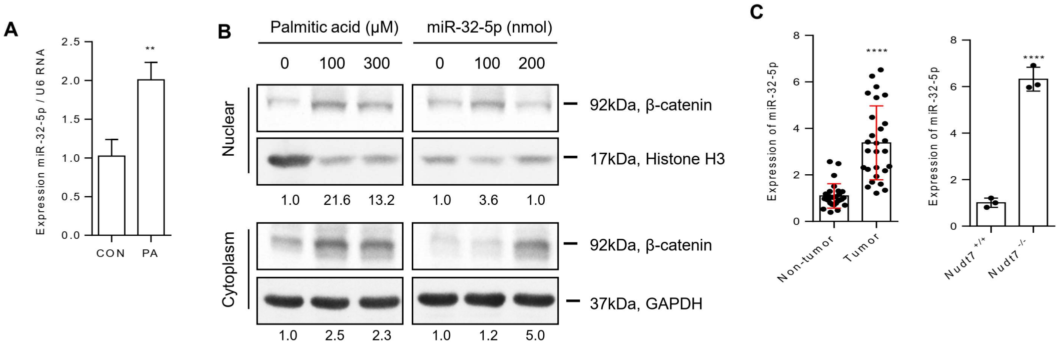

Figure S7. Palmitic acid induced expression level of miR-32-5p and an increased level of miR-32-5p activates Wnt/β-catenin signaling. (**A**) The expression level of miR-32-5p in Caco2 cells treated with or without palmitic acid (PA) was analyzed by real-time PCR (n = 3). *U6* was used as an endogenous control (**B**) The expression levels of β-catenin was analyzed using immunoblotting in Caco2 cells treated with PA or miR-32-5p mimic. Histone H3 or GAPDH were used as a loading control for nuclear protein or total protein lysate. (**C**) The expression level of miR-32-5p in CRC tumor compared with normal (n = 27) or in *Nudt7*$^{-/-}$ colon compared with *Nudt7*$^{+/+}$ colon (n = 3) were analyzed by real-time PCR. *Rn18s* was used as an endogenous control.

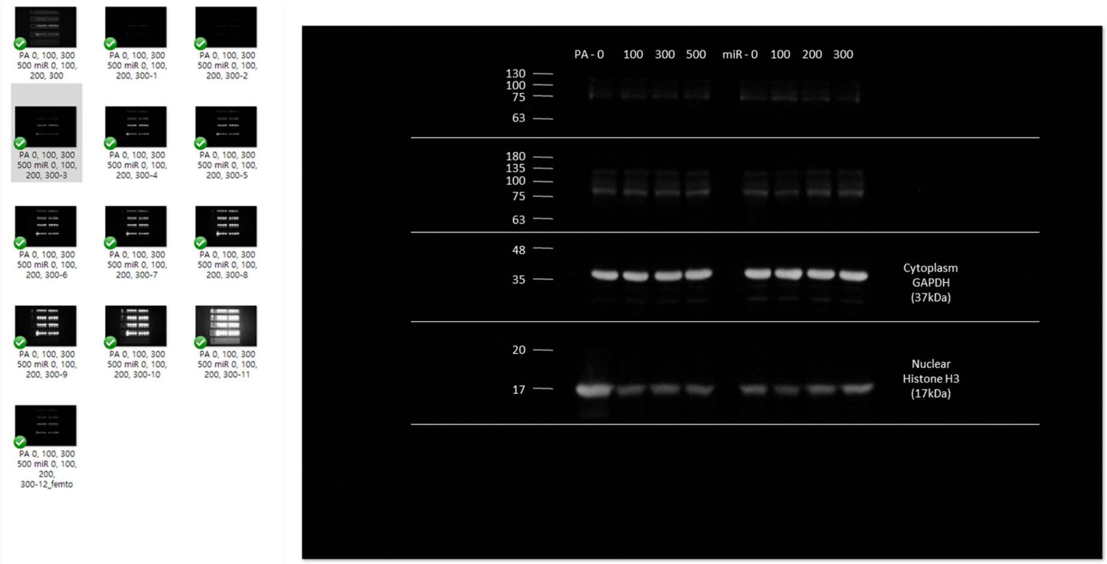

Uncropped images for Figure S7.

Table S1. Lipidomics analysis of CRC using gas chromatography/mass spectrometry (GC/MS). Palmitic acid is indicated in red.

Non-Tumor	Carbon	%	Tumor	Carbon	%
Indole	8	4.267	Palmitic acid	16	3.411
Palmitic acid	16	1.885	cis-Vaccenic acid	18	3.031
Dodecanamide	12	1.850	Dodecanamide	12	2.700
Oleic acid	18	1.694	Stearic acid	18	2.293
Palmitaldehyde	16	1.584	1-Hexadecene	16	1.234
ether, methyl 1-octadecenyl	19	1.438	Tetradecanal	14	1.140
9-Octadecenal	18	0.855	Linoleic acid	18	1.083
Coprosterol	27	0.746	Coprosterol	27	1.073
Palmitamide	16	0.659	ether, 1-hexadecenyl methyl	17	0.427
Squalene	30	0.365	Palmitamide	16	0.414
Tetradecanal	14	0.336	Palmitaldehyde	16	0.400
Linoleic acid	18	0.330	Ether,methyl 1-octadecenyl	19	0.380
Stearic acid	18	0.307	8-Octadecenoic acid	18	0.307
Tetradecane	14	0.276	cholest-7-en-3-ol, (3.beta.,5.alpha.)-	29	0.275
Arachidonic acid methyl ester	21	0.272	Arachidonic acid methyl ester	21	0.212
Docosane	22	0.244	1-Methoxy-1-hexadecene	17	0.200
18alpha-Oleanane	30	0.232	Arachidonic acid	20	0.175
1-Methoxy-1-hexadecene	17	0.222	Ether,methyl 1-octadecenyl	19	0.175
1-Hexadecene	16	0.167	Docosane	22	0.172
Eicosane	20	0.160	Squalane	30	0.167
Salicylaldehyde azine	14	0.134	Cyclotetradecane	14	0.145
2-Methyl-3-phenyl-1H-indole	15	0.129	2-Methyl-3-phenyl-1H-indole	15	0.140
2-Methyl-1-anthracenamine	15	0.126	Tetradecane	14	0.116
Oxyquinoline	9	0.124	Oleic acid	18	0.105
Tetradecyl trifluoroacetate	16	0.099	Squalene	30	0.094
Petroselaidic acid	18	0.077	Methostenol	28	0.081
DITRIDECYL PHTHALATE	34	0.072	trimethyl(4-tert-butylphenoxy)silane	13	0.074
Heneicosane,11-(1-ethylpropyl)-	26	0.062	Hexadecanoic acid,1,1'-[1-(hydroxymethyl)- 1,2-ethanediyl ester	35	0.065

Table 2. List of cell lines used in this study.

Cell line	KRAS	TP53	BRAF	PIK3CA	PTEN	MSI	Note
CaCo2	wt	p.E204X	wt	wt	wt	MSS	In this study / GSE97023
CL-34	wt	p.S127P; p.K382fs	p.V600E	wt	wt	MSI	GSE97023
Co115	wt	wt	p.V600E	wt	p.E157fs; p.R233X	MSI	GSE97023
Colo205	wt	p.Y107fs; p.Y103fs	p.V600E	wt	wt	MSS	GSE97023
Colo320	wt	p.R248W	wt	wt	wt	MSS	In this study / GSE97023
HT29	wt	p.R273H	p.V600E; p.T119Sc	wt	wt	MSS	In this study / GSE97023
KM12b	wt	p.P72fs; p.H179R	p.P403fs	wt	p.G129X; p.K267fs	MSI	GSE97023
NCI-H508	wt	p.R273H	p.G596R	p.E545K	wt	MSS	GSE97023
RKO	wt	wt	p.V600E	p.H1047R	wt	MSI	GSE97023
SW48	wt	wt	p.R347Xc	p.G914Rc	wt	MSI	GSE97023
V9P	wt	p.G245D	wt	wt	wt	MSS	GSE97023
WiDr	wt	p.R273H	p.V600E; p.T119Sc	wt	wt	MSS	GSE97023
CL-40	p.G12D	p.R248Q	wt	wt	wt	MSS	GSE97023
Colo678	p.G12D	wt	wt	wt	wt	MSS	GSE97023
EB	p.G12D	wt	wt	p.E545K	wt	MSS	GSE97023
IS1	p.G12D	p.Y163H	wt	wt	wt	MSS	GSE97023
IS3	p.G12D	p.Y163H	wt	wt	wt	MSS	GSE97023
LS174T	p.G12D	wt	p.D211Gc	p.H1047R	wt	MSI	In this study / GSE97023
SNU-407	p.G12D	wt	p.R726C	p.H1047R	wt	MSI	In this study
SNU-C2A	p.G12D	p.R141Y	wt	wt	wt	MSI	In this study
TC71	p.G12D	p.C176Y; p.R213X	wt	p.R88Qc	p.R233X	MSI	GSE97023

Table S3. List of real-time PCR primer used in this study.

Group	Gene Name	Forward	Reverse
Peroxisome genes (Human) http://www.peroxisomedb.org/	*ABCD1*	ATGCAAAGGAAGGGCTACTC	CGTCCTTCCAGTCACACATAG
	ABCD2	AAGAGAAGGAGGATGGGATG	GGTACATTCATCCAGCAAGG
	ABCD3	TGTCAGTCGCCCTTTCTTAG	CTATTCGACCCAGAGCTTGA
	ABCD4	TGAGCATCTTCGGGTATTTC	TCTGCATGTGCTTGAACCTA
	ACAA1	TAGCAGGTGGCATCAGAAATG	CTCCATCAAGCGCGAAGTAATA
	ACAD11	GCTGGTGTTTCCCAGGATTA	CAGCTCCATTGTTGCGATTG
	ACBD5	AATTGCTGCGTGTCATAGGT	TTTGGCGTTTGGAGTAGAAG
	ACOT1	GGTGACCAAAGATGGCTATG	TGACCTACCAGGAACAGGAA
	ACOT12	AGCACAAGCATGGAGATCAG	GTGGAGAAAGCCACACTAACA
	ACOT2	CTTGGTGGGCAGTCCTATTATC	CCCAAGTGTTTGTGGAAGAAAG
	ACOT4	CCACGTTGGCTCTAGCTTATT	GCATGTAGCATACGGCTTCT
	ACOT8	GGACCCTAACCTCCAAAAGA	ATCTGTTTGGGCTCCATTCT
	ACOX1	CAGGAATTACCGTTGGTGAC	TTCACCTGGGCATACTTCAT
	ACOX2	ACATGGCAAGAACAGCCTAC	TCATAACAGCCAAGTGCTGA
	ACOX3	TTGCTCTGACCGAATTAAGC	CGAAATCAGGGGAATGTATG
	ACSF3	ACACGTACAGGGAGCTTTATTC	GTTAGCGCATAGGAAGGAGAC
	ACSL1	ACGAAGATCCGCACTACTTG	CAAGGGCCATTATTTGACAC
	ACSL3	AAGCTTGCTAGGGGGAAATA	ACCAACAGGACAGCAGAAAC
	ACSL4	GCAGAGTACCCTGAAGGATTTG	CGTTGGTCTACTTGGAGGAATG
	ACSL5	TACCTGGGTTCCTGTCTCTT	GATGATCCACTCTGGCCTATTC
	ACSL6	TATCCGCCTTCTCTCAGATG	CTGGCTGAAGATCTTGTCGT
	AGPS	GTTGGCAGCTGGAGAAGATA	CCAAGGAGCAGAAGTCTCAA
	AGXT	GGTCCATGAGCAAGGATATGT	CAGAGATGACCAGTGTGAGTG
	ALDH3A2	CATGCTGGATGAGGCCTATATT	AGTGGCTGAATGGTGAGAAC
	AMACR	GCTGGCCACGATATCAACTAT	CAAAGTCAGCCAGGAGATTCA
	BAAT	GCTGAAGAGACATGGAAGAA	GCACAGCACAGAGGAGAATAG
	CAT	AATCCATTCGATCTCACCAA	GGTCGAAGGCTATCTGTTCA
	CRAT	AGACAAGGTGAACCGGGATTCCG	GGCTGCGGTACACGTCTTCTGA
	CROT	GCACTTCAGCTGGCCTATTA	ACTGCTTCAACTGTGCATGA
	DAO	GTTCCTAATCTCGGGCTACAAC	AACCAGCCATAGCCGTAATC
	DDO	CTGACGTGGTTCTGGGATTT	GGCATTCACATTTCAGGGTTG
	DECR2	ATACGGTGATTGCCAGTAGG	TCGGACGTCCATAGAGAGAG
	DNAJC10	CTTCAGTGGTCTCCCTTACAC	GATGACACCACGGAGAATAGAA
	DNM1L	GCAAAGGATCATTCAGCACT	CAGGCAACCTTTTACGAAGA
	ECH1	AAGAGGAATGCCATGAACAA	AACATTTTTCCTGCACCAGA
	EHHADH	TCTAGAAAGGGGCAAGGTCT	TTCACAGAGCACATCAGGAA
	EPHX2	CATCTGCTCCTCCCGAAATAG	CTTGAGAGAGGCCCAGTTTATC
	FAR1	GAGCTACCGGTTTTCTAGGG	GTGGTGTCTGTCCAGCTTTC
	FAR2	AGCAACAGCTTCACATCACA	TTAAAAGCCGATTCATGAGC
	FIS1	GACAAGGCCATGAAGAAAGA	GGATTTGGACTTGGACACAG
	GNPAT	TTTTTGTGCGCCCATCCTTAG	AAAACATCACGTAGGAAGCGAAAG
	GSTK1	CCTGTGCCGGTATCAGAATATC	GAGGCTTGTTTCCACTGTCT
	HACL1	ATGCTGGTACTTTCGGAACA	GAAAACCCAAATGCACTGTC
	HAO1	AGGCAGAGAAGATGGGCTACAA	TTTTCATCCTGAGTTGTGGCGG
	HAO2	GGAGATCAGTGCCCCTATTT	GCAAATGTGCTGGTGATGTA
	HMGCL	AAGGGCATTCAGAAGTTTCC	GGCAGCTCCAAAGATGACTA
	HSD17B4	GGCTTTGTCACGAGAGTTGT	CAGGAGTCATTGGGTGATTC
	IDE	CAAGTCAGCTGGTTCGGTATAG	GCTTTGCATGTCTGTTTGGTAG
	IDH1	CTAAGGGTTGGCCTTTGTATCT	GGGACTTGTACTGCTTGTCATA
	IDI1	CACCGAAAATAAGCTTCTGC	GCTGGATTGCTTAATGGATG
	IDI2	AGTCACGTTTCCTGGGTATTT	CACTCCGATGGCATCCTTT
	MLYCD	ACATCCAGGCAATCGTGAAG	CTGGGTCAAGCTGATGGAATAA

	Mosc2	ACCTGGGATGAACTCCTAATTG	AGTGGCTGTTTCCTGTCTATG
	MPV17	GCCAAACTACAGCGGGATTA	GGACAGGTAGGAGTTCCAGATA
	MVK	AGAGCAAGTGGAGAAGCTAAAG	CGGCAGATGGACAGGTATAAG
	NOS2	GTCAGAGTCACCATCCTCTTTG	GCAGCTCAGCCTGTACTTATC
	NUDT12	AGGAGGAAGATGGATTGGTTGCCT	CAGAAGGGCTGGCATAGGAGGA
	NUDT7	CTAAGGCCCGCTTAAGAAAGTA	CGGACGGTGAACAACAAATG
	PAOX	GGGAGTACCTCAAGAAGGAGAT	CCAGGTTGAAGAAGGAGTTCAG
	PECI	GACAGGGCAACATTTCATACAC	CTTGGCTGGGCTCATTATCT
	PECR	CTTGGTGAACAATGGAGGAG	GGAGCTGTAAACTGCTTTGC
	PEX1	TTCCTTAGGCGTATCCTCCTT	ATTCCTAAGTCCTGCAACAAGAG
	PEX10	CTCTCAGATGTGGCCTACTTT	CTGGATGATGCTGACGTACT
	PEX11A	GCTGCTCACCACTACTACTATTC	CCTGGGATGCTGATTTCTCTT
	PEX11B	TGCTAGACGTGGTCAGAAATG	CCAGGGATAGATTAGGGTGAGA
	PEX11G	GTTGGTGGAGTTCTGGTTGA	GGTCATCAAAGAGTCGCAAGA
	PEX12	CTTGCAGTTCCTTGACTGGT	TTGGGTAAGAGGGGAGAATC
	PEX13	TGGTCCTTACCTCATTTGGA	ACGGCAGCAAAATCATATTC
	PEX14	CCCTCACCTCATATCTCAGC	GGGGAGCAGGTATTTCTTGT
	PEX16	GAGCCTCCTGAGTGACAGAA	GCGGTCATAGAAAGGAGAGC
	PEX19	GGAGAGTGGGCAGTGATATG	CATGCTGGAGTTCTGAAGGT
	PEX26	TGGATGTACTTCAGGCCATT	ACAGGAACTTGTGGGAGACA
	PEX3	GAAATCTCGTTGAGCAGCAT	CCTGCACTGCTAATGGAGTT
	PEX5	TACAGCAGCAGGGTACATCA	CCCAGAAATCGACATCAGAC
	PEX5L	GTTCCACCTGAGTGGAGAATTTA	GAGGCGGTTCCATAGTGAATAG
	PEX6	AAACTGCAGGCCATCTTCTCCCGG	AGCCATCACACGGGCATCCTCA
	PEX7	CATGTCCTCATCACCTGTAGTG	CCTCCTGAGCGTGTTCTTTAT
	PHYH	GCTTTCGGAATGAGTTTGAA	TGGACCTTCGTGATCATCTT
	PIPOX	GAGAGAAGGTGGTGGAGATAAAC	GCTGTGATGACCAAGCTCTTA
	PMVK	TCAGCGGCAAGAGGAAATC	CCTGAGCATACTGTTCCTTGAG
	PRDX1	CGCACCATTGCTCAGGATTA	CCAACAGGGAGGTCATTTACAG
	PRDX5	GGATGTTCCAAGACACACCT	CCAGTCACAAAGGCATCATT
	PXMP2	TTTGTCAGCACTTGGGAACT	CCTGTGAAGAAGAACCCGTA
	PXMP3	GAATGCGAAGAGTGCAAACAG	CTGAGTAAACTGGGACCAAACT
	PXMP4	TGCCCTGCAGTCCTACATAC	TGTTGATCTGGCTGTTGATG
	RHOC	TGTCATCCTCATGTGCTTCTC	CTTGCCTCAGGTCCTTCTTATT
	SCP2	AGGGAAGCCTTGGAATAAAA	TCCAGCATACCCAAACATCT
	SLC22A5	CTCTCAGGGACGATTTGAAGAG	CTCACTCGGGTCAAAGATAGTG
	SLC25A17	CTGCTGCTCCAATTTTGTCT	CCTGCAACAAACCCAACTAC
	SLC27A2	CTCTTGCCTTGCGGACTAAA	CCGAAGCAGTTCACCGATATAC
	SOD1	GTGCAGGGCATCATCAATTTC	GGCCTTCAGTCAGTCCTTTAAT
	SOD2	GGAGATGTTACAGCCCAGATAG	CGTTAGGGCTGAGGTTTGT
	TRIM37	GGGACCAGCATTGGTACATTA	CTGAGTTCGAGACAGCTCAATAG
	XDH	AGCCTCTCGCCATCTTTATTC	TCTTTCAGCCTCAGCAACTC
Lipid metabolism (Human) http://amigo.geneontology.org/	*ABCA1*	GGTGGTGTTCTTCCTCATTACT	CCGCCTCACATCTTCATCTT
	ACACA	GCAGGTCACACGTCTCTTTAT	CCAGCCTGTCATCCTCAATATC
	ACSL1	GACGAGCCCTTGGTGTATTT	TTCATAGGGTTGGTCTGGTTTC
	AGPAT1	GAGGGAACGAGAAACCACAA	TCTTGGTAGGAGGACATGACTA
	CD36	CATTGGTGATGAGAAGGCAAAC	CACCACACCAACACTGAGTAA
	FASN	CTAGGTTTGATGCCTCCTTCTT	GATGGCTTCATAGGTGACTTCC
	LDLR	CTCCCGCCAAGATCAAGAAA	GTTTGGAGTCAACCCAGTAGAG
	PPARG	GCCTGCATCTCCACCTTATTA	ATCTCCACAGACACGACATTC
	SCD1	ACAACTACCACCACTCCTTTC	GGAGACTTTCTTCCGGTCATAG
	VLDLR	CAGGAAGATTGGCTTAGAGAGG	GCTTAGATCGGCCCAGAATAG
Mitochondria (Human)	*MCAD*	TGCTGGTGCTGTTGGATTAG	TGGTGCTCTACAAGTAGCTTTC
	NDUFA8	GACCTGGGAGAACTGTCAAAG	CGGTCTTGGTCTTGAGTGATAG
	SDHA	AGAGGGAGGCATTCTCATTAAC	ACCGAGACACCACATCTCTA

	UQCR2	CTCAGCAGCCATTTGATGTTTC	TGTGGCCTGGGAGATAGTATAA
CRC targe (Mouse) Ref. 26	Ccne1	CTGGATGTTGGCTGCTTAGA	TCTATGTCGCACCACTGATAAC
	Bmi1	CCAGACCACTCCTGAACATAAG	GACGGGTGAGCTGCATAAA
	Ccl2	CTCACCTGCTGCTACTCATTC	ACTACAGCTTCTTTGGGACAC
	Ccnd1 (β-Catenin target)	CAGAGGCGGATGAGAACAAG	GAGGGTGGGTTGGAAATGAA
	Cd16	CAAGCCTGTCACCATCACTG	GTGTCCACTGCAAACAGGAG
	Cd206	AGTGGCTTTGGTTGAACGAC	CCAAAGGCCCGAAGATGAAG
	Cd44 (β-Catenin target)	GAACCAGGACAGTGGAGTGA	GCAGACGGCAAGAATCAGAG
	F4/80	TGTACGTGCAACTCAGGACT	GTGGGACCACAGAGAGTTGA
	Il1b	CCACCTCAATGGACAGAATATCA	CCCAAGGCCACAGGTATTT
	Klf4	CCCTTCGGTCATCAGTGTTAG	GGACCGCCTCTTGCTTAAT
KrasG12D target (Mouse) Ref. 25	Aqp8	CTGTGTGTATGGGTGCTGTC	TTCATGCAGGCTCCAGAGAT
	Cdkn2a	GCTCTGGCTTTCGTGAACAT	GTGAACGTTGCCCATCATCA
	Fgf8	TTGGGAAAGCGGCTATTTGG	TCCTCGTGTGTTCCACTGAT
	Pdx1	AAGAGGACCCGTACTGCCTA	TTCAACATCACTGCCAGCTC
	Qpct	ACTCTTAACCCGGAAGCGAA	CTCCCACAAACACTCTGCTG
	Qsta1	AGCCATGGACAAGACTACCT	TCAGAAGGCTGGCATCAAAC
	Slc16a9	GCAGCTTGCCAAGAGGAAAT	TGAATAGCGCGGAGAAGACT
	Slc26a3	CTGCCATTCACGTTCTGGTT	AGACCGACTCCAGGACTTTG
	Ttr	CATGAATTCGCGGATGTGGT	CCGTGGTGCTGTAGGAGTAT
β-Catenin targ (Mouse)	Myc	CGCCTACATCCTGTCCATTC	AAGCTGTTCGAGTTTGTGTTTC
	Mmp14	CATCATTGAGGTGGATGAGGAG	CCATGGCGTCTGAAGAAGAA
	Nos2	ACAACTACCACCACTCCTTTC	GGAGACTTTCTTCCGGTCATAG
Endogenous control	Human *RN18S*	CTGAGAAACGGCTACCACATC	GCCTCGAAAGAGTCCTGTATTG
	Human *ACTB*	AGGCACCAGGGCGTGAT	GCCCACATAGGAATCCTTCTGAC
	Mouse *Rn18s*	CCAGTAAGTGCGGGTCATAAG	GGCCTCACTAAACCATCCAA

References

1. Park, S.; Oh, J.; Kim, Y.I.; Choe, S.K.; Chun, C.H.; Jin, E.J. Suppression of ABCD2 dysregulates lipid metabolism via dysregulation of miR-141:ACSL4 in human osteoarthritis. *Cell Biochem. Funct.* **2018**, *36*, 366–376, doi:10.1002/cbf.3356.
2. Schirripa, M.; Zhang, W.; Yang, D.; Cao, S.; Okazaki, S.; Loupakis, F.; Berger, M.D.; Ning, Y.; Miyamoto, Y.; Suenaga, M.; et al. NOS2 polymorphisms in prediction of benefit from first-line chemotherapy in metastatic colorectal cancer patients. *PloS one* **2018**, *13*, e0193640, doi:10.1371/journal.pone.0193640.
3. Chen, L.D.; Liu, Z.H.; Zhang, L.F.; Yao, J.N.; Wang, C.F. Sanggenon C induces apoptosis of colon cancer cells via inhibition of NO production, iNOS expression and ROS activation of the mitochondrial pathway. *Oncol. Rep.* **2017**, *38*, 2123–2131, doi:10.3892/or.2017.5912.
4. Dabbeche-Bouricha, E.; Hadiji-Abbes, N.; Abdelmaksoud-Damak, R.; Alaya, N.; Ayadi, W.; Charfi, S.; Khabir, A.; Sellami-Boudawara, T.; Mokdad-Gargouri, R. Quantitative measurement of iNOS expression in melanoma, nasopharyngeal, colorectal, and breast tumors of Tunisian patients: comparative study and clinical significance. *Tumour Biol.* **2016**, *37*, 5153–5164, doi:10.1007/s13277-015-4303-4.
5. Fransen, K.; Elander, N.; Soderkvist, P. Nitric oxide synthase 2 (NOS2) promoter polymorphisms in colorectal cancer. *Cancer Lett.* **2005**, *225*, 99–103, doi:10.1016/j.canlet.2005.02.006.
6. Reddy, B.S.; Hirose, Y.; Cohen, L.A.; Simi, B.; Cooma, I.; Rao, C.V. Preventive potential of wheat bran fractions against experimental colon carcinogenesis: implications for human colon cancer prevention. *Cancer Res.* **2000**, *60*, 4792–4797.
7. Puglisi, M.A.; Cenciarelli, C.; Tesori, V.; Cappellari, M.; Martini, M.; Di Francesco, A.M.; Giorda, E.;

Carsetti, R.; Ricci-Vitiani, L.; Gasbarrini, A. High nitric oxide production, secondary to inducible nitric oxide synthase expression, is essential for regulation of the tumour-initiating properties of colon cancer stem cells. *J. Pathol.* **2015**, *236*, 479–490, doi:10.1002/path.4545.
8. Skrzycki, M.; Majewska, M.; Podsiad, M.; Czeczot, H. Expression and activity of superoxide dismutase isoenzymes in colorectal cancer. *Acta Biochim. Pol.* **2009**, *56*, 663–670.
9. Bamodu, O.A.; Yang, C.K.; Cheng, W.H.; Tzeng, D.T.W.; Kuo, K.T.; Huang, C.C.; Deng, L.; Hsiao, M.; Lee, W.H.; Yeh, C.T. 4-Acetyl-Antroquinonol B Suppresses SOD2-Enhanced Cancer Stem Cell-Like Phenotypes and Chemoresistance of Colorectal Cancer Cells by Inducing hsa-miR-324 re-Expression. *Cancers* **2018**, *10*, doi:10.3390/cancers10080269.
10. Janssen, A.M.; Bosman, C.B.; Kruidenier, L.; Griffioen, G.; Lamers, C.B.; van Krieken, J.H.; van de Velde, C.J.; Verspaget, H.W. Superoxide dismutases in the human colorectal cancer sequence. *J. Cancer Res. Clin. Oncol.* **1999**, *125*, 327–335, doi:10.1007/s004320050282.
11. Nozoe, T.; Honda, M.; Inutsuka, S.; Yasuda, M.; Korenaga, D. Significance of immunohistochemical expression of manganese superoxide dismutase as a marker of malignant potential in colorectal carcinoma. *Oncol. Rep.* **2003**, *10*, 39–43.
12. Mohr, A.; Buneker, C.; Gough, R.P.; Zwacka, R.M. MnSOD protects colorectal cancer cells from TRAIL-induced apoptosis by inhibition of Smac/DIABLO release. *Oncogene* **2008**, *27*, 763–774, doi:10.1038/sj.onc.1210673.
13. Wang, F.; Wu, J.; Qiu, Z.; Ge, X.; Liu, X.; Zhang, C.; Xu, W.; Hua, D.; Qi, X.; Mao, Y. ACOT1 expression is associated with poor prognosis in gastric adenocarcinoma. *Hum. Pathol.* **2018**, *77*, 35–44, doi:10.1016/j.humpath.2018.03.013.
14. Konno, H.; Minamiya, Y.; Saito, H.; Imai, K.; Kawaharada, Y.; Motoyama, S.; Ogawa, J. Acquired xanthine dehydrogenase expression shortens survival in patients with resected adenocarcinoma of lung. *Tumour Biol.* **2012**, *33*, 1727–1732, doi:10.1007/s13277-012-0431-2.
15. Chen, W.C.; Wang, C.Y.; Hung, Y.H.; Weng, T.Y.; Yen, M.C.; Lai, M.D. Systematic Analysis of Gene Expression Alterations and Clinical Outcomes for Long-Chain Acyl-Coenzyme A Synthetase Family in Cancer. *PloS one* **2016**, *11*, e0155660, doi:10.1371/journal.pone.0155660.
16. Tang, Y.; Zhou, J.; Hooi, S.C.; Jiang, Y.M.; Lu, G.D. Fatty acid activation in carcinogenesis and cancer development: Essential roles of long-chain acyl-CoA synthetases. *Oncol. Lett.* **2018**, *16*, 1390–1396, doi:10.3892/ol.2018.8843.
17. Kuwata, H.; Hara, S. Role of acyl-CoA synthetase ACSL4 in arachidonic acid metabolism. *Prostaglandins Other Lipid Mediat.* **2019**, *144*, 106363, doi:10.1016/j.prostaglandins.2019.106363.
18. Benjamin, D.I.; Cozzo, A.; Ji, X.; Roberts, L.S.; Louie, S.M.; Mulvihill, M.M.; Luo, K.; Nomura, D.K. Ether lipid generating enzyme AGPS alters the balance of structural and signaling lipids to fuel cancer pathogenicity. *Proc. Natl. Acad. Sci. USA* **2013**, *110*, 14912–14917, doi:10.1073/pnas.1310894110.
19. Hlavata, I.; Mohelnikova-Duchonova, B.; Vaclavikova, R.; Liska, V.; Pitule, P.; Novak, P.; Bruha, J.; Vycital, O.; Holubec, L.; Treska, V.; et al. The role of ABC transporters in progression and clinical outcome of colorectal cancer. *Mutagenesis* **2012**, *27*, 187–196, doi:10.1093/mutage/ger075.
20. Lauer, C.; Volkl, A.; Riedl, S.; Fahimi, H.D.; Beier, K. Impairment of peroxisomal biogenesis in human colon carcinoma. *Carcinogenesis* **1999**, *20*, 985–989, doi:10.1093/carcin/20.6.985.
21. Yeh, C.S.; Wang, J.Y.; Cheng, T.L.; Juan, C.H.; Wu, C.H.; Lin, S.R. Fatty acid metabolism pathway play an important role in carcinogenesis of human colorectal cancers by Microarray-Bioinformatics analysis. *Cancer Lett.* **2006**, *233*, 297–308, doi:10.1016/j.canlet.2005.03.050.
22. Abu-Remaileh, M.; Bender, S.; Raddatz, G.; Ansari, I.; Cohen, D.; Gutekunst, J.; Musch, T.; Linhart, H.; Breiling, A.; Pikarsky, E.; et al. Chronic inflammation induces a novel epigenetic program that is conserved in intestinal adenomas and in colorectal cancer. *Cancer Res.* **2015**, *75*, 2120–2130, doi:10.1158/0008-5472.CAN-14-3295.
23. Luo, W.; Qin, L.; Li, B.; Liao, Z.; Liang, J.; Xiao, X.; Mo, Y.; Huang, G.; Zhang, Z.; Zhou, X.; et al. Inactivation of HMGCL promotes proliferation and metastasis of nasopharyngeal carcinoma by suppressing oxidative stress. *Sci. Rep.* **2017**, *7*, 11954, doi:10.1038/s41598-017-11025-2.
